# A leaky voltage sensor domain of cardiac sodium channels causes arrhythmias associated with dilated cardiomyopathy

**DOI:** 10.1038/s41598-018-31772-0

**Published:** 2018-09-14

**Authors:** Adrien Moreau, Pascal Gosselin-Badaroudine, Aurélie Mercier, Bettina Burger, Dagmar I. Keller, Mohamed Chahine

**Affiliations:** 1 CERVO Research Center, Institut universitaire en santé mentale de Québec, Quebec City, QC G1J 2G3 Canada; 2 Department of Biomedicine, University Hospital Basel, Basel, Switzerland; 3 Cardiology and Emergency Departments, University Hospital Zurich, Zurich, Switzerland; 4 Department of Medicine, Université Laval, Quebec City, QC G1K 7P4 Canada

## Abstract

Dilated cardiomyopathy (DCM) is a structural heart disease that causes dilatation of cardiac chambers and impairs cardiac contractility. The *SCN5A* gene encodes Na_v_1.5, the predominant cardiac sodium channel alpha subunit. *SCN5A* mutations have been identified in patients with arrhythmic disorders associated with DCM. The characterization of Na_v_1.5 mutations located in the voltage sensor domain (VSD) and associated with DCM revealed divergent biophysical defects that do not fully explain the pathologies observed in these patients. The purpose of this study was to characterize the pathological consequences of a gating pore in the heart arising from the Na_v_1.5/R219H mutation in a patient with complex cardiac arrhythmias and DCM. We report its properties using cardiomyocytes derived from patient-specific human induced pluripotent stem cells. We showed that this mutation generates a proton leak (called gating pore current). We also described disrupted ionic homeostasis, altered cellular morphology, electrical properties, and contractile function, most probably linked to the proton leak. We thus propose a novel link between *SCN5A* mutation and the complex pathogenesis of cardiac arrhythmias and DCM. Furthermore, we suggest that leaky channels would constitute a common pathological mechanism underlying several neuronal, neuromuscular, and cardiac pathologies.

## Introduction

Dilated cardiomyopathy (DCM) is a structural heart disease that causes dilatation of cardiac chambers and impairs cardiac contractility, eventually leading to heart failure. DCM is characterized by left ventricular enlargement (>117%) associated with systolic dysfunction (ejection fraction < 50%)^1–4^. Familial DCM is suspected in 20 to 48% of all DCM cases^5^ and is associated with mutations of genes encoding a number of cardiac-specific structural proteins, including sarcomeric proteins, cytoskeleton proteins, sarcomere-associated intermediate filaments, and nuclear lamina proteins^1^. The cardiac arrhythmias associated with DCM have also been linked to mutations of the *SCN5A* gene that encodes the Na_v_1.5 sodium channel, which is responsible for the initiation and propagation of cardiac action potentials^2,6–16^. *SCN5A* has since been identified as the sixth causative gene for familial DCM^5^. The underlying causes of DCM in *SCN5A*-mutated patients are not well understood. However, we have recently characterized the functional properties of five novel Na_v_1.5 mutations (Na_v_1.5/R219H, R222Q, R225W, R225P, and R814W) in familial DCM patients^11,17^. We previously used heterologous expression systems (Xenopus oocytes and tsA201 cells) to show that these mutations result in abnormal cation flows or “gating pore currents” through the channels’ voltage sensor domains (VSD)^11,17,18^. Gating pores are created by mutations in the VSD of voltage-gated ion channels (VGIC) and result in cation leaks through the usually non-conductive VSD^17–23^. In the present study, we used cardiomyocytes derived from patient-specific induced pluripotent stem cells (hiPSC-CM) to evaluate molecular and functional alterations in a cellular model harboring a gating pore. Gating pore currents most probably contributes to the morphological changes, impaired myocardial function, and cardiac arrhythmias observed in patients with DCM.

## Results

### Clinical phenotype of the index patient

We have been following a family for over 10 years. The index patient was diagnosed at age 29 with a clinical phenotype of complex arrhythmias associated with DCM (Table S1). Genetic testing revealed that the patient carries the Na_v_1.5/R219H mutation^11^. An implantable cardioverter defibrillator (ICD) was used to control ventricular tachycardia. The last echocardiography in January 2015 revealed the presence of DCM with a mild eccentric dilatation of the left ventricle (LV), a mild decrease in systolic function (LVEF: 49%) associated with general hypokinesia, mild mitral regurgitation, and severe enlargement of the left atrium (LA) (Fig. S1, Table S1). Over the course of 6 weeks, the patient neglected to take established therapies consisting of an ACE-inhibitor, a diuretic, and a beta-blocker, which led to a decreased LV systolic function (LVEF: 39%).

### Generation and cardiomyocyte differentiation of control and patient hiPSCs

**Figure 1:**
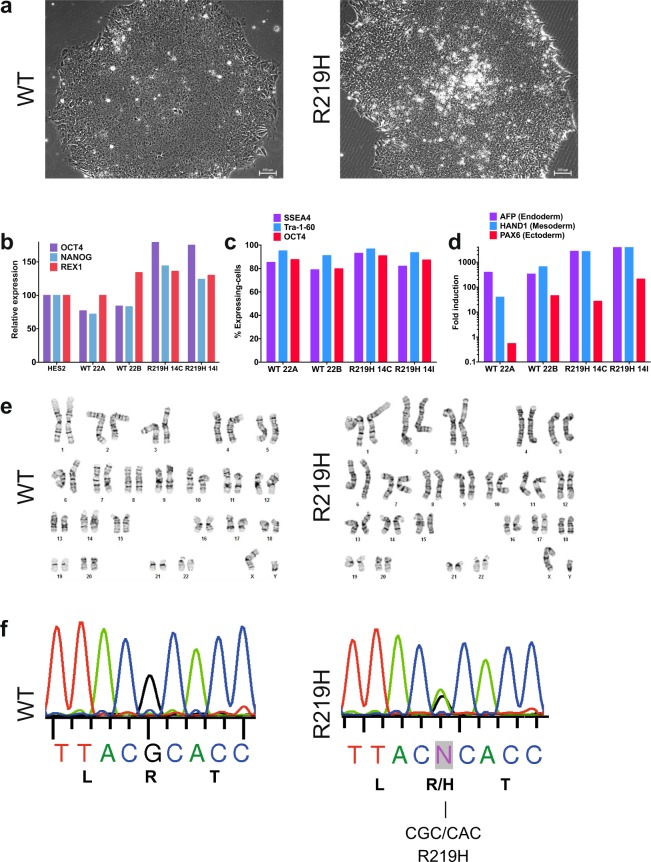
hiPSC characterization, quantification of pluripotency and differentiation markers. (**a**) Example of hiPSC WT and R219H colonies (x100). (**b**) Relative gene expression of pluripotency markers (OCT4, NANOG and REX1) assessed by qRT–PCR. The gene expression was normalized to the expression measured in human embryonic stem cells reference standards (HES2 cells). (**c**) Flow cytometry quantification of cells expressing the pluripotency–associated proteins (SSEA4, Tra–1–60, OCT4). (**d**) After embryoid body formation, the induction of germ lineage–specific markers was evaluated by qRT–PCR and compared with the same non–differentiated hiPSCs. The AFP, HAND1 and PAX6 genes were used as markers of endoderm, mesoderm and ectoderm. (**e**) The karyotype of hiPSC was evaluated by G–banding analysis. Both WT (left) and R219H (right) demonstrated normal karyotype. (**f**) The presence of the R219H point mutation in Na_v_1.5 was confirmed in genomic DNA of hiPSC cells from the patient while the mutation is absent in hiPSC from the healthy control father.

To investigate the pathological consequences of gating pore currents, we generated human hiPSCs from the *SCN5A*-DCM index patient and from his genetically and clinically unaffected father as a control. hiPSCs and hiPSC-CMs were characterized as schematically illustrated in Fig. S2. A multiscale analysis was performed to provide a complete characterization. Briefly, the morphology, electrical activity, and contractile function of the hiPSC-CMs were the main parameters considered in this study. hiPSCs were generated using the non-integrating Sendai virus method and were subsequently characterized (Fig. 1a–f). hiPSCs exhibited a typical round shape when grown as colonies (Fig. 1a) and expressed pluripotency markers such as OCT4, NANOG, REX1, SSEA4m and Tra-1–60, which were measured by qRT-PCR or fluorescence activated cell sorting (FACS) (Fig. 1b–c). Following embryoid body formation, the hiPSCs expressed higher levels of endoderm (AFP), mesoderm (HAND1), and ectoderm (PAX6) markers (Fig. 1d). The hiPSCs had normal karyotypes (Fig. 1e). The hiPSCs were differentiated into cardiomyocytes (hiPSC-CMs) using a variant of the Wnt-signaling pathway procedure^24,25^ (Fig. S2). The presence of the mutation was confirmed by sequencing both DNA and RNA (cDNA) (Figs 1f, S3a,b). Cardiac differentiation resulted in spontaneously beating cells (Videos S1 and S2). Differentiation efficacy was verified notably by analyzing the expression of cardiac markers by immunofluorescence (IF) (see below). qRT-PCR experiments also showed that the differentiation protocols resulted in the expression of GATA4 and cardiac troponin T (cTnT), two known cardiac markers (Fig. S3c). Western blotting revealed an increase in the expression of Na_v_1.5 channels as well as the cardiac nature of the cells (Fig. S3d). The current clamp technique was used to record the spontaneous electrical activity of hiPSC-CMs obtained using the differentiation protocol (Fig. S3e).

### Na_v_1.5 alpha and gating pore characterization of patient specific hiPSC-CMs

**Figure 2:**
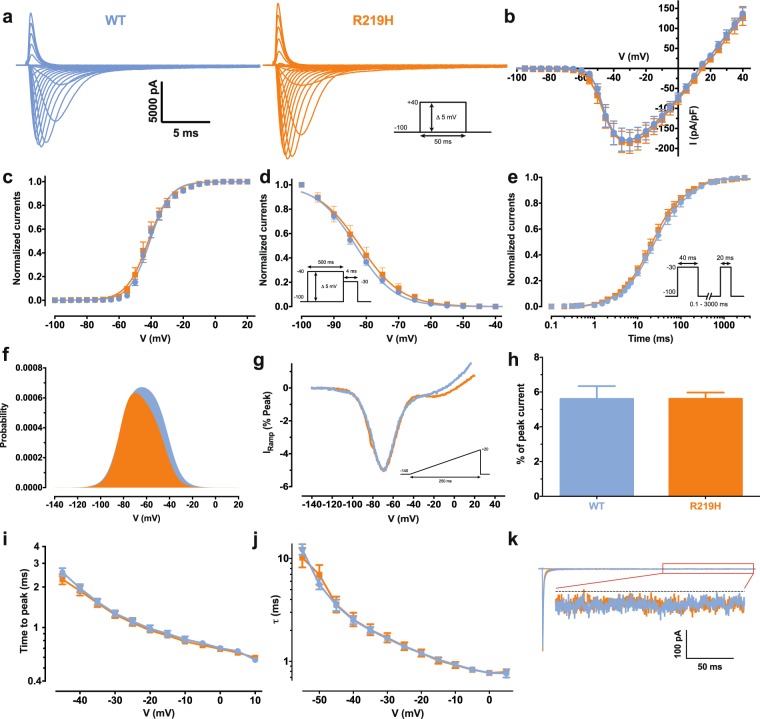
Biophysical characterization of the Na_v_1.5 channels from WT and patient specific hiPSC-CMs. The data of the Na_v_1.5 WT channel are indicated by blue symbols, those for the R219H mutant channel by orange symbols. (**a**) Representative whole–cell current traces of the WT and mutant channels. Currents were elicited using a voltage–clamp protocol where depolarizing pulses were applied for 50 ms from −100 to + 40 mV in 5 mV increments (see protocol in inset). (**b**) Current density–voltage (I–V) relationship of the Na_v_1.5/WT and R219H channels. (**c**) Voltage–dependence of steady–state activation of the WT and mutant channels. Activation curves were generated using a standard Boltzmann distribution [G(V)/G_max_ = 1/(1 + exp(−(V −V_1/2_)/k))] to give the V_1/2_ and k values listed in Table S2 of the article. (**d**) Steady state inactivation of the WT and mutant channels. Inactivation currents were obtained by applying conditioning pre–pulses to membrane potentials ranging from a holding potential of −100 to −40 mV for 500 ms in 5–mV increments and were then measured using a 4–ms pulse to −30 mV at each step (see protocol in inset). The recorded inactivation values were fitted to a standard Boltzmann equation [I(V)/I_max_ = 1/(1 + exp((V − V_1/2_)/k)) + C] to give the values listed in Table S5 of the article. (**e**) Recovery from fast inactivation was obtained using a two–pulse protocol at + 30 mV to obtain maximal activation (see protocol in inset). The time constants listed in Table S2 of the article were obtained using a two–exponential function: (A_fast_(1 − exp(−t/τ_fast_)) + A_slow_(1 − exp(−t/τ_slow_)) + C). (**f**) The overlap between activation and inactivation defines the window current. The predicted window current was obtained using the following equation: (1/(1 + exp((V_1/2activation_–V)/k_activation_)) × ((1–C)/(1 + exp((V − V_1/2inactivation_)/k_inactivation_)) + C). (**g**) Ramp protocols (see protocol in inset) were imposed to study the window current. As predicted in A, the window currents of the mutant channels is not different from WT channels. (**h**) Histogram showing the peak window current normalized to the alpha peak current (% of peak current). (**i**) The times to peak of the WT and mutant channels were used to evaluate the activation kinetics. The times to peak were measured on the same current traces used to construct the I–V relationship. (**j**) The time constants of fast inactivation decay were plotted as a function of voltage for the WT and mutant channels. The time constants were obtained using a simple–exponential function: (A_fast_(exp(−t/τ) + C). (**k**) Representative current traces after a −30 mV depolarizing pulse indicating a similar persistent Na^+^ current from Na_v_1.5 WT and R219H channels. The persistent Na^+^ current is measured at the end of the 400–ms depolarizing pulse to give the values listed in Table S2.

We first recorded Na^+^ currents from WT and heterozygous R219H hiPSC-CMs and assessed their biophysical properties using the patch clamp technique (Fig. 2a–k, Table S2). The biophysical properties of Na^+^ currents recorded from R219H myocytes, including activation (Fig. 2c), inactivation (Fig. 2d), recovery from fast inactivation (Fig. 2e), calculated window current (Fig. 2f), ramp elicited current (Fig. 2g), the presence of a persistent Na^+^ current (Fig. 2h,k) and current kinetics (time to peak, current decay) (Fig. 2i,j) were not altered by the presence of the Na_v_1.5/R219H mutation

**Figure 3:**
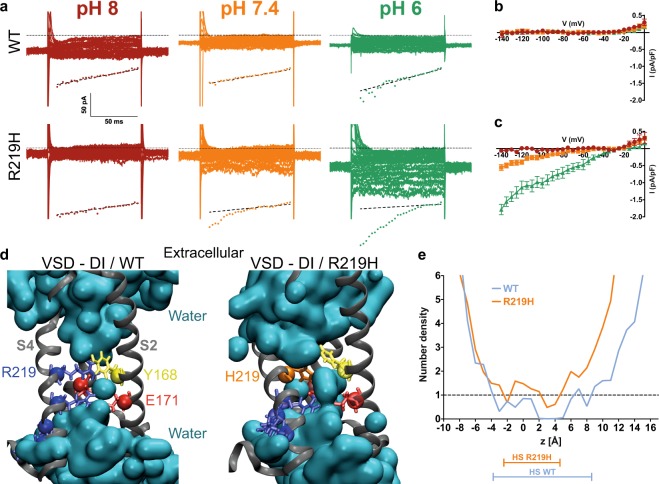
The Na_v_1.5/R219H mutation opens a proton specific gating pore. (**a**) Gating pore currents were recorded from a holding potential of −80 mV using a voltage-step protocol from −140 to 0 mV in 5-mV increments. The top panels show examples of raw traces of gating pore currents for each hiPSC cell line (WT or Na_v_1.5/R219H hiPSC-CMs). The currents are also plotted as a function of voltage in the bottom panels. Linear non-specific leaks are indicated by dotted lines. The patient specific hiPSC-CM carrying the Na_v_1.5/R219H mutation generates a gating pore current that is not observed with the WT hiPSC-CM. The gating pore is open at hyperpolarized potentials and specifically conducts protons. (**b**,**c**) Current density-voltage relationships of gating pore currents recorded for the WT hiPSC-CM (**b**) and R219H hiPSC-CM (**c**) are shown (n = 6 for WT and n = 7 for R219H). (**d**) Structural models of the relaxed domain I (DI) VSD of the WT Na_v_1.5 (left) and the R219H mutant (right). The VSD protein backbone is represented as a grey ribbon. For the purpose of clarity, the S1 segment of the VSD has been removed. The gating charges of S4 and the counter charges of S2 and S3 are shown using standard colors (positive charges in blue, negative charges in red, aromatic residues in yellow, and the histidine 219 in orange). In the middle panel, the water-accessible volume is shown as a transparent cyan surface. (**e**) Water density profiles along the main axis of the VSD WT (blue) and R219H (orange). The histograms were built using a 1–Å grid, and the averages were calculated from the last 10 ns of the trajectories. 0 corresponds to the position of the Cα of Y168 of S2. The hydrophobic septum (HS) for each VSD is determined by a water density below 1.

Interestingly, compared to their WT counterparts, hiPSC-CMs expressing Na_v_1.5/R219H channels exhibited a gating pore current at hyperpolarized potentials (at pH 7.4, −140 mV: 0.0009 ± 0.02 pA/pF, n = 6 for WT cells vs. −0.55 ± 0.09 pA/pF, n = 7 for R219H cells) (Fig. 3a). Voltage-dependence was recorded using 5-mV voltage steps from −140 mV to 0 mV from a holding potential of −80 mV (Fig. 3b,c). The gating pore current, which was absent in WT hiPSC-CMs, was pH_o_-dependent (increased at lower pHs) and was larger at more hyperpolarized voltages (for R219H hiPSC-CMs at −140 mV, n = 7: −0.06 ± 0.03 at pH 8, −0.55 ± 0.09 at pH 7.4, and −1.79 ± 0.15 pA/pF at pH 6). The substitution of the first arginine with a histidine should create a shuttle that moved protons from the extracellular to the intracellular milieu. Molecular dynamic simulation experiments showed that the *in-silico* mutagenesis of the histidine at position 219 occurs in the gating charge transfer center (GCTC), a region that separates two water crevices where protons can shuttle into the cell (Figs 3d, S4). During the activation process, each arginine of the S4 segment is expected to sequentially interact with the GCTC, forming a hydrophobic septum and preventing ions from crossing the membrane (Figs 3d,e and S4). When this first arginine is mutated (and replaced with a histidine), molecular dynamic simulations showed that integrity of the hydrophobic septum (HS) is partially affected and that the histidine may create a bridge between the two water crevices (Figs 3d,e and S4). This possibility was also supported by the water density profile, which exhibited a larger HS for the WT VSD (Fig. 3e).

### Patient-specific hiPSC-CMs recapitulate cellular dilatation and altered sarcomeric organization

**Figure 4:**
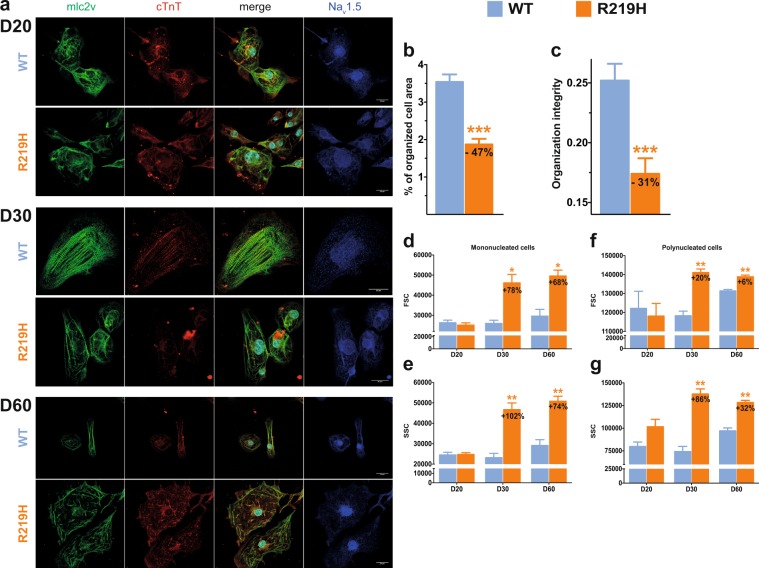
hiPSC-CM morphology and sarcomeric organization. The results for the WT hiPSC-CMs are indicated in blue, those for the R219H hiPSC-CMs are in orange. (**a**) Immunocytochemistry of the WT and Na_v_1.5/R219H hiPSC-CMs at D20 (top), 30 (middle) and 60 (bottom) of differentiation. The first column of each panel represents myosin light chain 2 v staining (mlc2v, green) and the second column represents cardiac troponin T staining (cTnT, red). The third column shows the merger of columns one and two with the DAPI nuclear staining. For the WT hiPSC-CMs, the nucleus cannot be clearly seen due to the confocal image. The nucleus is just below, at the limit of the confocal section. The last column of each panel represents Na_v_1.5 channels (blue). (**b**,**c**) Contractile structural organization characteristics studied using AutoTT software^54^. The cell area covered by the mlc2v contractile protein (**b**) and organizational integrity (**c**) were evaluated. (**d–g**) FACS technique revealed two distinct hiPSC-CM populations comprising mononucleated cells and polynucleated cells (that are described in Figure S7). Histograms summarizing the size (forward scatter, FSC) and granularity (side scatter, SSC) of hiPSC-CMs (WT and R219H) at D20, D30, and D60 of differentiation evaluated using the FACS technique.

To determine some aspects of the DCM phenotype of the index patient’s hiPSC-CMs, we evaluated the structural changes to contractile proteins and the distribution of Na_v_1.5 channels by IF (Figs 4, S5). We detected significant structural differences in the organization of myosin light chain 2 v (mlc2v) and troponin T (cTnT) between R219H and WT hiPSC-CMs (Fig. 4a–c). While the WT hiPSC-CMs exhibited a typical striation pattern, there was a prominent lack of normal organization in the R219H hiPSC-CMs. Interestingly, at D20 of differentiation (early stage of differentiation), the R219H hiPSC-CMs appeared to be unaffected and were very similar to the WT hiPSC-CMs (Fig. 4a). However, at D60 of differentiation (late stage of differentiation), the defects in the R219H hiPSC-CMs were much more pronounced (Fig. 4a). However, the organized areas in the WT and R219H myocytes were identical, as can be seen in the higher magnification images and in the organizational spacing (Fig S5a–c). We extended our evaluation of cellular striation using AutoTT software, which uses a fast-Fourier transform (FFT) approach (Fig. S6). AutoTT revealed that the cell surface covered by regular striation was decreased by 47% in Na_v_1.5/R219H hiPSC-CMs when compared to normalized WT hiPSC-CMs (100%) (Fig. 4b,c).

Since the clinical phenotype of our index patient included the development of DCM, a large-scale flow cytometric analysis of the size and granularity of the hiPSC-CMs was performed on two R219H clones. As expected, mononucleated and multinucleated hiPSC-CMs were detected (Figs 4d–g, S7a–c, Table S3). Interestingly, WT hiPSC-CMs exhibited a larger proportion of polynucleated cells (64.2 ± 3.8% of WT cells *vs*. 36.2 ± 1.2% of R219H cells), which is a marker of maturation^26,27^ (Fig. S7b). At D30 of differentiation, mononucleated and polynucleated R219H hiPSC-CMs were larger than their WT counterparts (Figs 4d,f, S7c), likely due to cellular dilatation, and exhibited noticeable granularity (Figs 4e,g, S7c), presumably due to the disorganization of contractile proteins. Like the IF observations, the FACS results showed that at D20 of differentiation, there were no statistically significant differences between WT and R219H hiPSC-CMs in terms of the proportion of polynucleated cells, cell size, or granularity (Figs 4d–g and S7b,c, Table S3). However, at D60 of differentiation, the differences in terms of the proportion of polynucleated cells, cell size, and granularity persisted (Figs 4d–g and S17b,c, Table S3).

### Patient-specific hiPSC-CMs exhibited altered action potentials and ionic homeostasis

**Figure 5:**
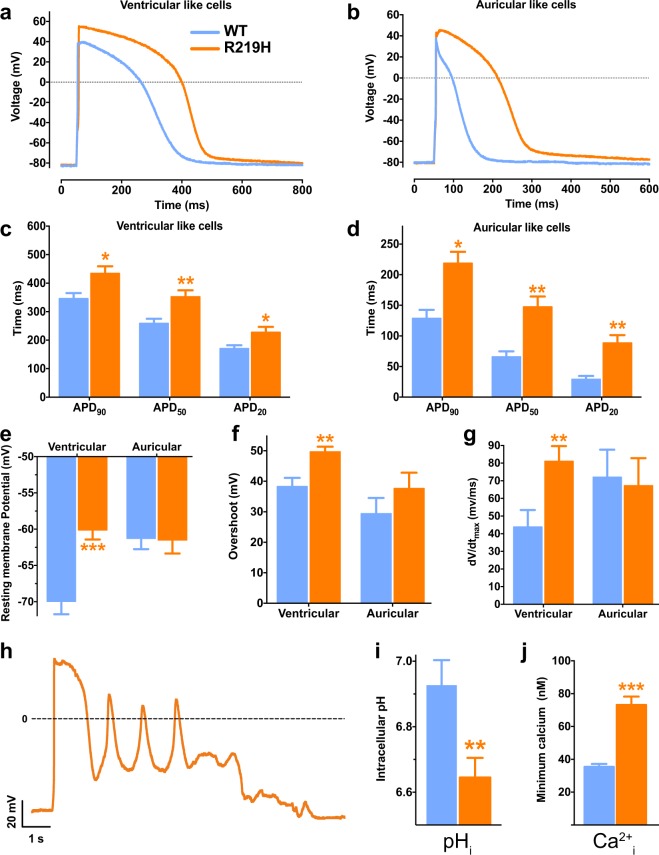
Electrophysiological properties of WT and R219H hiPSC-CMs. The results for the WT hiPSC-CMs are indicated by blue symbols and those for the R219H hiPSC-CMs by orange symbols. APs were recorded for both WT and R219H hiPSC-CMs using the current clamp technique (whole cell configuration) where APs were elicited using 3-ms pulses at a frequency of 1 Hz. (**a**,**b**) Examples of raw traces of APs of ventricular-like and auricular-like cells recorded from WT and R219H hiPSC-CMs. (**c**,**d**) Ventricular-like (**c**) and auricular-like (**d**) hiPSC-CMs harboring the Na_v_1.5/R219H mutation exhibit a prolonged AP duration (APD). (**e**) The Na_v_1.5/R219H mutation caused a depolarization of the resting membrane potential (RMP) measured just after reaching the whole cell configuration. (**f**) The overshoot, defined as the maximum potential reached during the AP, is slightly higher for ventricular-like cells carrying the Na_v_1.5 R219H mutation. (**g**) dV/dt, defined as the maximal upstroke velocity of the AP, is also slightly higher for ventricular-like cells carrying the Na_v_1.5/R219H mutation. (**h**) Arrhythmic events (early after depolarizations, EADs) were recorded in gap-free mode (current clamp) for a ventricular-like R219H hiPSC-CM. (**i**) R219H hiPSC-CMs have an acidic intracellular pH as measured using the BCECF-AM fluorescent probe (n = 34 and 60 for the WT and R219H hiPSC-CMs, respectively). (**j**) Intracellular calcium levels were measured using the fura-2-AM ratiometric fluorescent probe (n = 106 and 78 for the WT and R219H hiPSC-CMs, respectively). The asterisks indicate differences between WT hiPSC-CMs (*p < 0.05, **p < 0.01, ***p < 0.001). Detailed values and the number of experiments are reported in Table S3.

To get further insights into the pathogenic mechanisms underlying the clinical phenotype, the potential corollaries of the gating pore currents were evaluated on action potential (AP) recordings from WT and R219H hiPSC-CMs at 30 days of differentiation (Fig. 5). The AP duration (APD) was prolonged in two distinct DCM R219H hiPSC-CM clones compared to their WT counterparts (Fig. 5c,d, Table S4). The resting membrane potential (RMP) was depolarized in ventricular-like myocytes from the R219H patient (Fig. 5e, Table S4). AP upstrokes and overshoots were higher in magnitude (Fig. 5f,g, Table S4). qRT–PCR results showed that more depolarized RMPs and prolonged AP durations are not likely due to a decreased expression in voltage-dependent K_v_ channels (Fig. S8). Studying the effects of the proton gating pore on AP parameters at several stimulation frequencies revealed that the proton leak current was more deleterious at both low and high frequencies (APs were not successfully elicited at 2 Hz for the R219H hiPSC-CMs) (Fig. S9a–i and Table S4). Arrhythmic events (early or delayed after depolarizations, EADs/DADs) were also observed in both auricular-like and ventricular-like R219H hiPSC-CMs (Figs 5h and S9j). Indeed, two of the five ventricular-like hiPSC-CMs recorded in gap-free mode displayed arrhythmic events while none of their WT counterparts did (n = 6).

Due to the H^+^ specificity of the gating pore current, we suspected an impact on ionic homeostasis and evaluated several physiological ion concentrations. Intracellular pH (pH_i_) and calcium (Ca^2+^_i_) levels were quantified in hiPSC-CMs loaded with pH dye (BCECF) or Ca^2+^ dye (Fura-2), respectively. R219H hiPSC-CMs were more acidic than their WT counterparts (pH_i_ = 6.93 ± 0.08, n = 34 for WT *vs*. 6.65 ± 0.06, n = 60 for R219H) (Fig. 5i), while “diastolic” Ca^2+^ levels were significantly higher in R219H hiPSC-CMs ([Ca^2+^] = 35.4 ± 1.8 nmol/L, n = 106 for WT *vs*. 73.1 ± 5.1 nmol/L, n = 78 for R219H) (Fig. 5j).

### Patient-specific hiPSC-CMs exhibit impaired contractility

**Figure 6:**
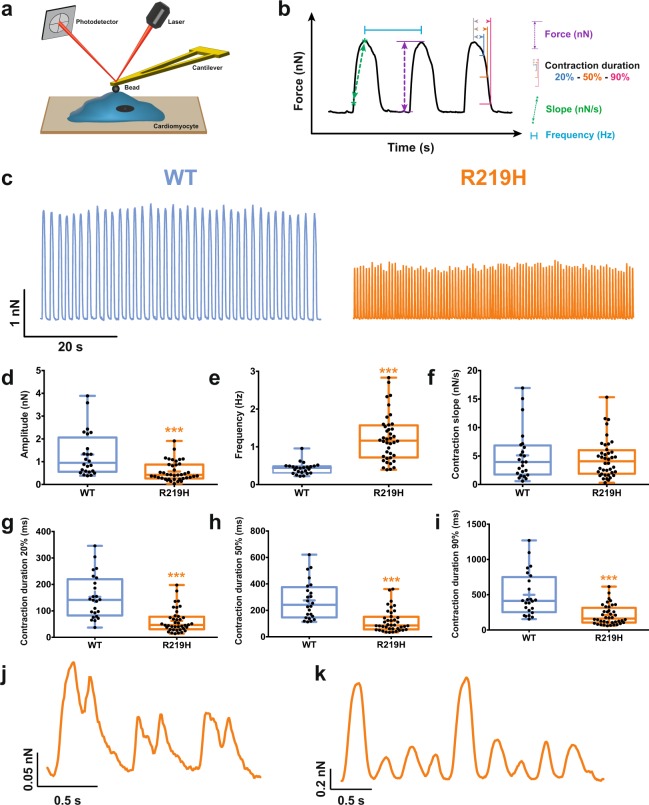
Evaluation of hiPSC-CMs spontaneous contractile function by atomic force microscopy. The results for the WT hiPSC-CMs are indicated by blue symbols, and those for the R219H hiPSC-CMs by orange symbols. (**a**) Schematic representation of the atomic force microscope experimental setup used for the evaluation of contractile function. (**b**) Example of a magnified raw trace showing the different parameters measured on each trace. The contraction slope, the amplitude (force) and duration are evaluated. The contraction frequency is also evaluated. (**c**) Representative raw traces of contractile functions monitored using an atomic force microscope (AFM) for both hiPSC-CMs. (**d**–**i**) Whisker box plot of all contractile parameters. The means of individual cells (black dots), 25^th^, 50^th^, and 75^th^ percentile quantiles (box) and the global mean (cross) are shown for each plot. The gating pore current caused a decrease in the contraction amplitude (**d**), an increase in the contractile frequency (**e**), and a shortening of the contraction duration (**g**–**i**). (**j–k**) Examples of chaotic contractions recorded for the R219H hiPSC-CMs. Asterisks indicate differences between WT hiPSC-CMs (***p < 0.001).

As impaired contractility is a characteristic trait of DCM^28^, we assessed the contractility of single myocytes by atomic force microscopy (AFM) (Fig. 6a–c, and Table S5). Remarkably, at 37 °C, the amplitude of contraction of single spontaneously beating R219H hiPSC-CMs was lower than that of WT hiPSC-CMs (1.31 ± 0.20 nN, n = 24 for WT *vs*. 0.57 ± 0.07 nN, n = 40 for R219H) (Fig. 6d, and Table S5). Their contraction frequency was higher (0.43 ± 0.03 Hz, n = 24 for WT *vs*. 1.23 ± 0.10 Hz, n = 40 for R219H) (Fig. 6e, and Table S5) while their contraction duration was shorter (contraction duration at 90% of relaxation: 499 ± 63 ms, n = 24 for WT *vs*. 210 ± 22 ms, n = 40 for R219H) than that of WT hiPSC-CMs (Fig. 6g–i, and Table S5). They also displayed chaotic contraction behavior (Fig. 6j,k). These modified contractile properties could be explained by alterations to the intracellular ionic homeostasis (Fig. 6i,j).

## Discussion

The worldwide prevalence of DCM has recently been recently estimated to be 1 in 250, indicating that DCM is an important public health issue^1^. *SCN5A* mutations are potentially involved in 3% of all DCM cases^1^. Although little is known about the pathological mechanism linking an ion channel to a structural heart defect, this would make *SCN5A* mutations the sixth leading cause of DCM. Interestingly, DCM patients harboring *SCN5A* mutations also suffer from various auricular and ventricular arrhythmias.

We recently used structural models of the four Na_v_1.5 VSDs incorporating all the mutations reported to cause cardiac arrhythmias associated with DCM in order to show that these mutations are located in close proximity to the VSD GCTC^12^. Interactions between the S4 segment and the GCTC are known to form a hydrophobic septum that separates water crevices spanning the extracellular and intracellular milieu^6,17,19,29,30^. Mutations disrupting these interactions might “connect” these water crevices and create a gating pore that would act as a pathway through the VSD by which cations could cross the cell membrane.

While gating pores are a well-established pathological cause of hypokalemic periodic paralysis (HypoPP)^6,22,31–36^, there is no proof of their pathological involvement in heart disease. We used patient-specific hiPSC-CMs derived from hiPSC to study the electrical, morphological, and contractile properties of a model harboring a gating pore to propose their potential pathological consequences. We showed that patient hiPSC-CM which display a gating pore, also display altered contractile protein structures, APs, and contractile functions. Results regarding AP parameters and contractile function might initially appear as divergent (AP lengthening while contraction duration is reduced). However, one might note that APD are analyzed for hiPSC-CM paced at a fixed frequency (1 Hz) while contraction duration is obtained under natural beating of hiPSC-CM (no pacing, 37 °C environment). To accurately compare APD and contraction duration, the values compared should take into account this different beating frequency (0.43 and 1.23 for WT and R219H respectively). Mean APD_90_ at 0.5 Hz for WT is 438 ms and mean APD_90_ at 1.3 Hz for R219H is 359 ms, thus indicating consistent data between electrical and contractile functions.

The pathological effects observed might also result from the altered ionic homeostasis probably caused by gating pores. Interestingly, the effect of intracellular pH on AP parameters are consistent with recent reports. Indeed, a mutation of the chloride-bicarbonate exchanger has recently been reported to be responsible for elevation in intracellular pH^37^. Using a zebrafish model and rabbit cardiomyocytes, the authors demonstrated a shortening in APD due to this pH_i_ increase, the exact opposite of our APD lengthening due to pHi decrease. Similarly, using rabbit and guinea pig ventricular myocytes, Saegusa and co-workers demonstrated that intracellular acidosis lengthen APD, notably through slowing ICaL inactivation kinetics, finally leading in an increase Ca^2+^ entry during the AP accompanied with increased diastolic Ca^2+^ levels^38^. Intracellular acidification can depolarize cardiac myocytes by blocking K_ir_ channels^39,40^. K_ir_ blocks have already been shown to depolarize cell RMPs and prolong AP durations^41,42^. Electrical disturbances caused by K_ir_ blocks is also a commonly accepted explanation for HypoPP pathogenesis, a gating pore-related pathology^39,43,44^. Depolarized RMPs may explain the premature ventricular depolarizations observed in our index patient. The significantly elevated intracellular Ca^2+^ levels (Fig. 5j) could be explained by a rise in both H^+^ and Na^+^ levels resulting from the activation of a Na^+^/H^+^ antiporter that causes the accumulation of Ca^2+^ *via* the reverse mode of the Na^+^/Ca^2+^ exchanger. The observed acidosis has also been reported to reduce the affinity of troponin C for Ca^2+^, impairing excitation-contraction coupling^45^. In addition, the Ca^2+^ overload could lead to incomplete cardiomyocyte relaxation and affect myofilament function^46–48^. Alterations to ionic homeostasis, including acidification, may also uncouple gap junctions (e.g., by changing the phosphorylation status) as already suggested for Cx40^49^ and Cx43^50^ and affect their integrity^51^. This may contribute to the diverse auricular dysfunctions (atrial fibrillation and flutter) and conduction disturbances seen in patients suffering from similar Na_v_1.5 VSD mutations^11^. The imbalance in ionic homeostasis may thus have many detrimental impacts on electrical activity, cellular structure and morphology, and cardiomyocyte contractility. All these mechanisms may explain the development of both DCM and arrhythmias.

The VSD is a specialized structure that senses voltage changes. It is common to all voltage sensitive proteins, including VGIC. Given the broad physiological roles and distribution of these proteins^6^, at least 69 other mutations that could result in the creation of a gating pore may contribute to the development of numerous neuronal, skeletal, and cardiac disorders (Fig. S10).

## Conclusion

The electrical, structural, and contractile disturbances we describe here could explain the cardiac arrhythmias associated with DCM observed in patients with similar clinical phenotypes. We suggest that this gating pore current should be systematically investigated to characterize similar *SCN5A* mutations with comparable clinical phenotypes. Our results also support the hypothesis that the gating pore current is pathological in nature and should thus be considered as a member of a large family of pathologies.

## Methods

Detailed descriptions are presented in the Online Supplemental Material.

### Production, derivation, culture, characterization, and differentiation of patient-specific hiPSCs

The local ethics committee of Basel University hospital (Universitätsspital Basel) approved the study protocol. The study was conducted according to the principle of the declaration of Helsinki. Signed informed consents were obtained for all cases. Skin biopsies and blood samples from the index patient and the healthy control (father, who does not harbor the R219H mutation) were collected. Both cell lines were reprogrammed and were characterized at the Center for Commercialization of Regenerative Medicine (CCRM, Toronto, ON, Canada) core facility using the OCT3/4, SOX2, KLF4, and C-MYC reprogramming factors and the non-integrating Sendai virus method.

### Patient-specific hiPSC differentiation

For differentiation purposes, hiPSC lines grown as colonies were adapted to MEF-free conditions. The hiPSCs were differentiated into cardiac myocytes based on recently published protocols^24,25^. The cells were exposed to a series of reagents in a time-controlled manner to induce differentiation. A schematic diagram of the protocol is shown in Fig. S2. The cells were allowed to recover for at least 6 days before beginning the experiments.

### Immunostaining

Seven days prior to the experiments, hiPSC-CMs were dissociated and were plated in Nunc^TM^ Lab-Tek^TM^ II CC2^TM^ chambers. The cells were fixed and permeabilized and were then incubated with primary and secondary antibodies. The cells were observed using a Zeiss LSM confocal microscope equipped with a 63x oil objective and the appropriate laser and filters.

### Western blots

Proteins were extracted from WT and Na_v_1.5/R219H hiPSC-CMs on D0 and D30 of differentiation as previously described^52^.

### Electrophysiology

Patch clamp experiments were conducted using an Axopatch 200B amplifier (Axon Instruments, Foster City, CA, USA) and standard protocols at room temperature at least 6 days following hiPSC-CM dissociation. Macroscopic Na^+^ currents were recorded using the whole cell configuration of the patch clamp technique. APs were evaluated using the whole cell configuration of the patch clamp technique (in current clamp mode). The gap-free mode was used to record spontaneous APs. For this purpose, electrical activity was recorded without intervention.

### Ca^2+^ and H^+^ concentration evaluations

The BCECF-AM (2′,7′-Bis-(2-carboxyethyl)-5-(and -6)-carboxyfluorescein, acetoxymethyl ester) ratiometric fluorescent probe was used to assess the intracellular pH (pHi) in hiPSC-CMs. The fura-2-AM ratiometric fluorescent probe was used assess intracellular Ca^2+^ (Ca^2+^i) in hiPSC-CMs.

### Atomic force microscopy (AFM)

hiPSC-CMs were maintained at 37 °C for the entire experiment in pre-warmed external current clamp solution (see Online supplement). Typically, 50 to 400 beats were collected for each cell, and statistics were calculated for the force (amplitude), rising slope, intervals between beats to obtain the frequency, and duration of each contraction (duration at 20%, 50%, and 90% of relaxation).

### Data analysis and statistics

The electrophysiological results were analyzed using Clampfit (pCLAMP v10.0; Molecular Devices) and custom-written MATLAB programs (The MathWorks Inc.). The AFM results were analyzed using custom-written MATLAB programs (The MathWorks Inc.). The confocal microscopic results were analyzed using ImageJ and AutoTT software. The AutoTT software was kindly provided by Dr. Long–Sheng (University of Iowa). Cell sorting results were analyzed using Diva software (BD FACSDiva^TM^) and Flowing software 2. Molecular dynamic simulation images were obtained using VMD software^53^. Results are expressed as means ± SEM. When indicated, a t test was performed using GraphPad prism software (GraphPad Software, Inc.). Differences were considered significant at a p < 0.05 (*), p < 0.01 (**), or p < 0.001 (***).

## Electronic supplementary material

Supplemental Data

Video WT

Video R219H
