# Supplementary material for: A leaky voltage sensor domain of cardiac sodium channels causes arrhythmias associated with dilated cardiomyopathy
Source: Sci Rep. 2018 Sep 14;8:13804. doi: 10.1038/s41598-018-31772-0 (PMC6138662; doi:10.1038/s41598-018-31772-0)

A leaky voltage sensor domain of cardiac sodium channels causes arrhythmias associated with dilated cardiomyopathy

Adrien Moreau, Ph.D., Pascal Gosselin–Badaroudine, Ph.D., Aurélie Mercier, Ph.D., Bettina Burger, Ph.D., Dagmar I. Keller, M.D., and Mohamed Chahine, Ph.D.

Online Supplement

Supplemental methods:

Production of patient–specific hiPSC, derivation, culture, characterization and differentiation

Skin biopsies or blood sample from the patient and the healthy control (father, non R219H mutation carrier) were collected with informed consent and standardized approved protocols. Two human induced pluripotent stem cells (hiPSC) lines were investigated in the study: hiPSC WT and R219H from the patient previously described and his healthy father[1]. Both cell lines were reprogrammed and characterized at the center for commercialization of regenerative medicine (CCRM, Toronto, ON, Canada) core facility which used the reprogramming factors: OCT3/4, SOX2, KLF4, and C–MYC *via* the non–integrating Sendai virus methods. Control hiPSCs were reprogrammed from human $CD34^+$ peripheral blood mononuclear cells, while hiPSC R219H was reprogrammed from patient human dermal fibroblasts. During the hiPSC reprograming process and their characterization, hiPSC cells were cultured on mouse embryonic fibroblasts (MEFs) and regularly passaged every 5–7 days using collagenase IV (1 mg/ml, 8 minutes, 37°C). Cells were cultured in hiPSC growth medium: DMEM/F12 supplemented with 20% knockout serum replacement, 2 mmol/L L–glutamine, 1% non–essential amino acid (NEAA), 0.1 mmol/L 2–mercaptoethanol, 10 ng/mL bFGF (freshly added) and 50 000 u/ml of penicillin, 50 000 µg/ml of Streptomycin. For characterization purposes hiPSC

WT and R219H were grown on MEFs. MEFs were always removed prior to analysis. The characterization consisted of the evaluation of the expression of pluripotency–associated proteins, the evaluation of the gene expression of pluripotency markers, the ability of germ layer differentiation, the evaluation of the identity between initial samples and hiPSC and the evaluation of the karyotype. The expression of surface markers (SSEA4 and Tra–1–60) and intracellular markers (OCT4) was evaluated using flow cytometry and immunofluorescence. Gene expression of pluripotency markers (OCT4, NANOG and REX1) was evaluated using qRT–PCR. The germ layer differentiation ability was evaluated by the formation of embryonic bodies followed by qRT–PCR for the evaluation of AFP (endoderm marker), HAND1 (mesoderm marker) and PAX6 (ectoderm marker). The identity of hiPSC cells with original cells was verified by short tandem repeat (STR) analysis and the karyotype was carried out to rule out eventual chromosomal abnormalities (> 3–10 Mb).

Patient specific hiPSC differentiation

For the purpose of differentiation, hiPSC lines grown as colonies were adapted to MEF free conditions. Feeder free hiPSC were cultured on hESC qualified Matrigel with mTeSR1 medium. hiPSCs were typically passaged at 1:8 ratio every 5 to 6 days using Dispase (1mg/ml, 2–3 min, 37°C) and were differentiated into cardiac myocytes based on the recently published protocols[2,3]. Cells were exposed to a series of reagents in a time–controlled manner to induce differentiation; a schematic diagram of the protocol is shown in Figure S2. Briefly, 5 days prior the initiation of differentiation, hiPSC colonies were dissociated into single cells using TrypLE express (7–8 min, 37°C), seeded onto hESC qualified Matrigel and cultured in mTeSR1 medium (medium changed daily). At day 0, the differentiation is initiated using RMPI 1640 medium supplemented with B27 supplement minus insulin (1X) and 6 μmol/L of CHIR99021 (LC laboratories). At day 2, the medium was replaced by RMPI 1640 supplemented with B27 supplement minus insulin (1X). At day 3, the medium is replaced by RMPI 1640 supplemented with B27 supplement minus insulin (1X) and 2 μmol/L of Wnt–C59 (LC laboratories). At day 5 and 7, the medium was replaced by RMPI 1640 supplemented with B27 supplement minus insulin (1X). From day 9, the medium was replaced every other

day by RMPI 1640 supplemented with B27 supplement (1X). Beating cells appears from days 7 to 12. Accordingly to the recently published data, this process is expected to result in a high efficiency of hiPSC differentiation into cardiac myocyte with more than 80% of cTnT positive cells in each well[2,4]. Unless stated otherwise, for all experiments, beating cells were dissociated after at least 24 days of differentiation into single cells using TrypLE express for 10 minutes and plated onto 0.1% gelatin coated plastic dishes in RPMI 1640 supplemented with B27 supplement (1X). Cells were then allowed to recover for at least 6 days before beginning the experiments.

Genomic DNA and total RNA extraction

The genomic DNA from hiPSCs cultured on Matrigel was extracted using a lysis buffer composed of (in mmol/L): 10 NaCl, 10 Tris–HCl, 10 EDTA and 0.5% SDS, pH 7.5. The DNA was then incubated overnight with proteinase K and precipitated using isopropanol.

Total RNA was extract from hiPSC or hiPSC–CM cells using Trizol kit (Sigma) was done according to the manufacturer's instructions. RNA integrity was evaluated by Gel red™ (Biotium) staining of 1% agarose gels. Total RNA was quantified by recording the optical density at 260 and 280 nm.

Reverse transcriptase

Based on total RNA extracted from hiPSC or hiPSC–CM, reverse transcriptase was performed using Transcriptor First Strand cDNA Synthesis kits (Roche) according to the manufacturer's protocol. 1 µg of total RNA was added to random hexamer primer and water to obtain a volume of 13 µL. This mixture was denatured for 10 min at 65°C. The reaction buffer, composed of RNase inhibitor (20 U final concentration), DNTP (1 mmol/L each), and Transcriptor Reverse Transcriptase (1 U final concentration), was added to the mixture to obtain a final volume of 20 µL. cDNA was synthesized at 50°C for 1 h. The enzymes were heat–inactivated at 85°C for 2 min. cDNA was stored at −80°C until used.

DNA and RNA (cDNA) sequencing

The *SCN5A* gene (DNA) and its coding sequence (cDNA) were amplified by PCR using primers listed in table S6 and sequenced using the Sanger method (Applied Biosystems 3730xl DNA Analyzer) at the "Plateforme de séquençage et de génotypage (CRCHUL)" http://www.sequences.crchul.ulaval.ca/ core facility.

Real–time polymerase chain reaction (RT–qPCR)

qPCR assays were done using SYBR green I detection dye on a LC480 platform (Roche) using vendor specifications. Primers listed in table S6 were designed using PerlPrimer v1.1.19 and Primer3. All qPCR samples were run at least in duplicate, and for each plate we applied a non–template control (NTC) and positive control for each primer pair to control every qPCR run. The qPCR reaction was carried out using an initial step of 5 min at 95°C to activate Taq polymerase, followed by 45 cycles consisting of 10 s at 95°C, 10 s at 58°C, and elongation at 72°C for 12 s. The qPCR efficiencies were obtained using series cDNA dilutions. The qPCR efficiency was calculated using the slope of the regression line, according to the equation E = 10 [–1/slope]. Analysis has been done with the lightcycler software LightCycler® 480 SW 1.5. Run–to–run variation has been adjusted using a known standard and quantification was corrected for efficiency. The specificity of the amplification for each run was controlled with a melting curve analysis, directly following the PCR by continuously reading the fluorescence while slowly heating the reaction from 65°C to 95°C.

Flow cytometry

hiPSC–CM were dissociated at indicated day of differentiation. Single cells were fixed 10 minutes using 4% paraformaldehyde in PBS. Fixed cells were then centrifuged 5 minutes at 200g and resuspended in PBS at 500 000 cells/ml. PFA fixed hiPSC–CM cells were analyzed on a FACS Aria II (BD Biosciences) with a 100 µm–size nozzle and a sheath pressure of 20 PSI. Forward–scatter (FSC) and side–scatter (SSC) data were obtained on cell populations positive for propidium iodide (PI) incorporation.

Immunostaining

Seven days prior to experiments, hiPSC–CM cells were dissociated and plated onto Nunc^{TM} Lab–Tek^{TM} II CC2^{TM}. The cells were fixed using 37°C PBS – 4% paraformaldehyde (PFA) – 4% sucrose solution for 20 minutes. Cells were then permeabilized for 30 minutes at room temperature using 0.1% triton in a PBS – 1% bovine serum albumin (BSA) – 5 % normal goat serum (NGS) solution. Primary anti–bodies to detect myosin–light chain–2v (mlc2v, 1:300, AP02680PU–N, Acris antibodies), cardiac troponin T (cTnT, 1:300, Ab10214, Abcam) and $Na_v1.5$ channels (1:300, AGP–008, Alomone labs) were incubated overnight at 4°C. Fluorophore conjugated secondary anti–bodies: Alexa Fluor® 488 goat anti rabbit (1:250, A11070, Life Technologies), Alexa Fluor® 594 goat anti mouse (1:250, A11005, Life Technologies) and Alexa Fluor® 647 goat anti guinea pig (1:250, A21450, Life Technologies) were incubated one hour at room temperature. The nucleus were labeled using DAPI (4',6–Diamidino–2–phenylindole dihydrochloride). Cells were observed on a Zeiss LSM confocal microscope with a 63x oil objective equipped with appropriate laser and filters.

Images of the mlc2v protein obtained with the confocal microscope were extracted and converted into 8–bit gray scale. Images were then studied using the AutoTT software to evaluate the organization spacing, the organization integrity and the cell area covered by contractile organization. The cell area covered by contractile organization was corrected for the size of each analyzed hiPSC–CM cell.

Western blot

Protein extractions were performed in WT and heterozygous R219H hiPSC–CM day 0 and day 30 as described previously[5]. Protein concentrations in the cleared lysates were determined using DC protein assay (Biorad). Equal amounts of total proteins (30 μg) were denatured in Laemmli 2x buffer (Sigma) for 30 min at 37°C, resolved on 10% SDS–polyacrylamide gel and blotted onto 0.45–μm Immobilon polyvinylidene difluoride (PVDF) membrane (Millipore). Membranes were blocked and subsequently incubated with anti–$Na_v1.5$ primary antibody (1:200, Alomone Labs) and anti–β–actin (1:20 000, Sigma) followed by anti–

rabbit or anti–mouse horseradish peroxidase–conjugated secondary antibodies (1:10 000, Jackson Laboratories). Proteins were visualized using a chemiluminescence detection kit (GE Healthcare).

Electrophysiology

Patch clamp experiments were conducted using an Axopatch 200B amplifier (Axon Instruments, Foster City, CA, USA), at room temperature at least 6 days after the hiPSC–CM dissociation. For all experiments the liquid junction potential between the patch pipette and the bath solution was not corrected. Pipettes were made from 8161 corning borosilicate glass capillaries and fire polished. For voltage–clamp experiments, pipettes were coated using HIPEC (Dow–Corning, Midland, MI, USA) to minimize electrode capacitance. Macroscopic Na^+ current were recorded using the whole cell configuration of the patch clamp technique. The patch pipettes were filled with a solution containing (in mmol/L): 105 CsF, 35 NaCl, 10 EGTA and 10 Hepes, pH was adjusted to 7.4 using 1N CsOH. The bath solution was composed of (in mmol/L): 105 NMDG, 35 NaCl, 2 KCl, 1.5 $CaCl_2$, 1 $MgCl_2$, 10 glucose, 10 Hepes, 10 TEA–Cl, 0.5 Nimodipine, pH was adjusted to 7.4 using methanethiosulfonic (MTS) acid. Voltage–clamp command pulses were generated by a microcomputer using pCLAMP software v10 (Axon Instruments). Na^+ currents were filtered at 5 kHz, digitized at 10 kHz, and stored on a microcomputer equipped with an AD converter (Digidata 1440A, Axon Instruments). P/4 leak subtraction was used prior to test pulses.

For gating pore current recordings, similar experimental set–up was used. P/4 leak subtraction was not used but linear leak subtraction was performed off–line at depolarized voltages where the gating pore is closed to eliminate inherent non–specific leaks. The patch pipets were filled with solution containing (in mmol/L): 105 CsF, 35 NaCl, 10 EGTA and 10 Hepes, pH was adjusted to 7.4 using 1N CsOH. The bath solutions were composed of (in mmol/L): 145 NMDG, 2 $Ca(OH)_2$ and either 20 Hepes (pH 8 / 7.4) or 20 MES (pH 6), pH was adjusted at 6, 7.4 or 8 using MTS acid.

Action potentials (APs) were evaluated using the whole cell configuration of the patch clamp technique (in current clamp mode). The gap–free mode was used to record spontaneous APs. For this purpose, the electrical activity was recorded without intervention. For other current clamp experiments, APs were elicited

using a 3–ms, 20 – 1500 pA rectangular current pulse injection at different frequency (0.5, 0.8, 1, 1.3, 1.5 and 2 Hz). The patch pipets (resistance 2 – 5 mΩ) were filled with a solution containing (in mmol/L): 10 NaCl, 122 KCl, 1 $MgCl_2$, 1 EGTA, 10 Hepes, pH was adjusted at 7.3 with KOH. The bath solution (external current clamp) was composed of (in mmol/L): 154 NaCl, 5.6 KCl, 2 $CaCl_2$, 1 $MgCl_2$, 8 Glucose, 10 Hepes, the pH was adjusted at 7.3 with 1N NaOH.

Ca^{2+} and H^+ concentration evaluation

The BCECF–AM (2',7' – Bis – (2 – carboxyethyl) – 5 – (and – 6) – carboxyfluorescein, acetoxymethyl ester) ratiometric fluorescent probe was used for intracellular pH (pHi) evaluation in hiPSC–CM. Cells were incubated for 15 minutes at 37°C with 5 μM of BCECF–AM in tyrode solution (in mmol/L: 140 NaCl, 5.4 KCl, 1.8 $CaCl_2$, 1.8 $MgCl_2$, 10 Glucose, 10 Hepes, pH 7.4 with Tris base). The pHi was evaluated on a Zeiss Observer D1 inverted microscope equipped with appropriate wheel filters and an Excite source of light. After recording the initial fluorescence (at both wavelength: 490nm and 440 nm), the fluorescence signal was calibrated to obtain the maximum and minimum fluorescence signal using high KCl solutions at pH 6 and 8 supplemented with 10 μmol/L nigericin and 10 μmol/L monensin. The high KCl solutions were composed of (in mmol/L): 145 KCl, 1.8 $CaCl_2$, 1.8 $MgCl_2$, 10 glucose and either 10 Hepes (pH 8) or 10 MES (pH 6). The pH was adjusted at 6 or 8 using Tris base. The calibration allows to calculate the pHi using the following equation:

$$pH = pK_a - \log\frac{(R - R_{min})}{(R_{max} - R)} \times \frac{F_A}{F_B}$$

Where: pK_a, is fixed to 7.0 according to the manufacturer's indication; R, is the ratio of fluorescence (F_{490}/F_{440}); R_{min}, is the minimum ratio of fluorescence (obtained with the High KCl pH 6 solution); R_{max}, is the maximum ratio of fluorescence (obtained with the high KCl pH 8 solution); F_A, is the fluorescence at 440 nm obtained using the High KCl pH 8 solution and F_B, is the fluorescence at 440 nm obtained using the High KCl pH 8 solution.

The fura–2–AM ratiometric fluorescent probe was used for intracellular Ca^{2+} ($Ca^{2+}i$) assessement in hiPSC–CM. The procedure used to measure $Ca^{2+}i$ is similar to the one used for the pHi evaluation. Briefly, hiPSC–CM cells were loaded for 30 minutes at 37°C with 5 µmol/L of fura–2–AM in tyrode solution. After measuring the fluorescence (at both wavelength: 340 and 380 nm) at 0.7 Hz for 2–3 minutes, the fluorescence signal was calibrated using two modified tyrode solutions (containing 0 mmol/L and 20 mmol/L Ca^{2+}) to reach the minimum and maximum fluorescent signals. Modified tyrode 0 Ca^{2+} solution contained (in mmol/L): 140 NaCl, 5.4 KCl, 1.8 $MgCl_2$, 10 Glucose, 10 Hepes, 10 EGTA, pH 7.4 with Tris base. Modified tyrode 20 Ca^{2+} contained (in mM): 120 NaCl, 5.4 KCl, 20 CaCl2, 1.8 $MgCl_2$, 10 Hepes, pH 7.4 with Tris base. 10 µmol/L of ionomycin was added to each calibration solutions. The calibration allows to calculate the [$Ca^{2+}{}_i$] using the following equation:

$$[Ca^{2+}] = K_d\, Q\, \frac{(R - R_{min})}{(R_{max} - R)}$$

Where: [Ca^{2+}], is the calcium concentration; K_d, is the Ca^{2+} dissociation constant fixed to 0.14 according to the manufacturer's indication; Q, is the ration of minimum fluorescence to maximum fluorescence at 380 nm; R, is the ratio of fluorescence (F_{340}/F_{380}); R_{min}, is the minimum ratio of fluorescence (obtained with the modified tyrode 0 mmol/L Ca^{2+}) and R_{max}, is the maximum ratio of fluorescence (obtained with the modified tyrode 20 mmol/L Ca^{2+}).

Atomic force microscopy (AFM)

hiPSC–CM were maintained at 37°C for the entire experiment in pre–warm external current clamp solution. To ensure the absence of transient thermal effects of the cantilever deflection, the experimental chamber was equilibrated for at least 20 minutes prior to any measurements. Single beating hiPSC–CM were interrogated by AFM (NanoWizard II AFM (JPK)) with a silicon nitride MLCT cantilever (spring constants ~0.01 N/m, Bruker AFM probes). The cantilever tip (8 ± 0.4–µm–diameter bead, Duke Standards™,

Thermo Scientific) was gently (300 pN) put in contact with the cell and remained in position without Z–piezo feedback for 2 min while the deflection data was collected at a sample rate of 1 kHz. Typically, 50 to 400 beats were collected for each cell, and statistics were calculated for the force (amplitude), the rising slope, the intervals between beats to obtain the frequency, and the duration of each contraction (duration at 20%, 50% and 90% of relaxation).

Molecular modeling

Using the procedure described in Moreau et al. 2015, models for the WT and R219H $Na_v1.5$ VSDs were generated[6]. Briefly, the high–resolution Na_vAb structure revealed in 2011[7] was used as template because, according to PSI–BLAST searches in the PDB database, Na_vAb's sequence is the most similar to $Na_v1.5$ domain investigated here. Pairwise alignments between the sequence of the first domain and the Na_vAb sequence were extracted from a multiple sequence alignment of the first 100 mammalian sequences homologous to Na_vAb according to a PSI–BLAST search of Na_vAb against NCBI's reference protein database. The first domain of the human $Na_v1.5$ appears in the alignment generated with Clustal Omega[8]. Standard MODELLER routines[9] were then used to generate comparative models of $Na_v1.5$ channel's DI VSD. Similarly to the models presented in Moreau et al. 2015, the S3 helix was surprisingly short in all models. We therefore imposed helicity on a larger part of the S3 segments in our MODELLER routines as described in Moreau et al., 2015[6].

In order to place the R219 residues in the GCTC, the pairwise alignments of the S4 segments used to generate the model was shifted by 9 residues toward the C–terminus. This 3–helical turn shift had the effect of placing the residue to be mutated in the GCTC. According to previous work, these would be the conformations in which the VSD would yield gating pore currents as a result of the R219H mutation[1,6,10]. Similar procedures were used in recent investigations focused on VSDs[6,11] and tend to result in more stable conformational states than steered molecular dynamics procedure used in our previous work[10].

The homology models were then inserted in a POPC bilayer and equilibrated under normal constant temperature and pressure conditions (298 K, 1 atm) in a 150 mM NaCl solution. The lipid tails were first

melted during the first nanosecond while restraining the position of the protein, the lipid head groups, the water molecules and the ions to their initial positions. Afterwards, only the protein was restrained for 2–ns. Those restraints were relaxed progressively for 6–ns. Lastly, a 100–ns unrestrained MD simulation of the entire VSD was conducted, enabling the system to relax. The MD simulations were carried out using the NAMD2 program. Langevin dynamics were applied to keep the temperature fixed (300ºK). The equations of motion were integrated using a multiple time–step algorithm. Short– and long–range forces were calculated every 1 and 2 time–steps, respectively, with a time step of 2.0–fs. The simulation used the CHARMM22–CMAP force field with torsional cross–terms for the protein and CHARMM36 for the phospholipids.

Mutant R219H VSD was created by introducing the mutation on the equilibrated WT VSD using MODELLER. It was equilibrated using the same procedure.

The simulations were made on the supercomputer Colosse, managed by Calcul Québec and Compute Canada. The operation of this supercomputer is funded by the Canada Foundation for Innovation (CFI), NanoQuébec, RMGA and the Fonds de recherche du Québec – Nature et technologies (FRQ–NT).

Supplemental figure legends:

Figure S1: Transthoracic echocardiography of the index patient

End–diastolic apical four chamber view of the heart of the index patient in 2009 (left) and in 2015 (right) indicating a mild dilatation of the heart chambers. Images show as indicated the right and left ventricles (RV, LV) and the right and left atria (RA, LA).

Figure S2: Experimental procedures

Schematic representation of experimental procedures used for the characterization of hiPSC–CM WT and R219H. Top panel represents a time line of conditions used for the cardiac differentiation of hiPSCs.

Bottom panel represents a time line of experiments performed on hiPSCs and hiPSC–CMs for their characterization.

Figure S3: hiPSC differentiate into hiPSC-CM

(a) The presence of the R219H point mutation in $Na_v1.5$ was confirmed in cDNA (RNA) from patient specific hiPSC–CM while the mutation is absent in hiPSC-CM from the healthy control father. **(b)** The differentiation process importantly increases the presence of $Na_v1.5$ cDNA (RNA) in both control and R219H cells. (**c**) Relative quantities versus RPL22 (internal for each hiPSC-CM lines) of GATA4 and cTnT transcripts evaluated by real–time reverse transcription polymerase chain reaction (qRT–PCR) using mRNA extract from hiPSC–CM at day 60 of differentiation. **(d)** The presence of $Na_v1.5$ protein in hiPSC–CM is confirmed by western blot experiments. $Na_v1.5$ is detected in both WT and mutant hiPSC–CM (D_{30}) while $Na_v1.5$ protein is not detected in hiPSC (D_0). The molecular weight is indicated in kDa. The β–actin expression appears stable. The full-length blot is provided figure S11. (**e**) The action potentials (AP) of the hiPSC–CM WT cells are indicated in blue (top), those for the R219H cells in orange (bottom). Based on action potential duration (APD), 3 types of AP were recorded from both WT and R219H hiPSC–CM. Dotted lines indicate the zero voltage.

Figure S4: 3D models of $Na_v1.5$/WT and R219H DI VSDs

Models of the DI VSD of $Na_v1.5$/WT (top) or R219H (bottom) were built based on previously published results[6]. The protein is shown as grey ribbons, and the water crevice is shown in blue. Positively charged residues in the S4 segment and residues making up the GCTC are highlighted. Positively charged residues are depicted in blue, negatively charged residues in red, aromatic residues in yellow, and the 219 histidine in orange. The complete VSD is shown (left) for each panel. A higher magnification of the GCTC is also shown to clearly see the interactions between the S4 segment and the GCTC (middle) as well as the hydrophobic septum (right) for WT and R219H VSDs. For purposes of clarity, the S1 segment has been removed in middle and right panels.

Figure S5: hiPSC–CM sarcomeric organization

(a) The detailed organization of mlc2v and cTnT or mlc2v and Na_v1.5 proteins is shown for hiPSC–CM WT (left) and R219H (right). **(b)** Schematic representation of the organization observed using the immunocytochemistry experiments. **(c)** The spacing of mlc2v proteins was evaluated using the AutoTT software[12] for both WT and R219H hiPSC–CM.

Figure S6: Structural analysis using the AutoTT software

Images from the immunocytochemistry experiments were further analyzed for contractile proteins organization. Top images (hiPSC–CM WT left and R219H right) are examples of grey scale confocal images of mlc2v proteins used in the AutoTT software[12]. The background is automatically removed and images are then skeletalized to detect structural organization. The area covered by structural organization is then calculated and off line corrected by the area of the cell to give a percentage of area covered by contractile organization. The organization is also analyzed with a Fast Fourier Transform (FFT) to obtain the spacing of contractile proteins and the integrity of organization.

Figure S7: FACS hiPSC-CM characterization

The FACS experiments were realized using propidium iodide to initially discriminate hiPSC–CM from debris. The frequency histogram indicate that hiPSC–CMs are populated of mononucleated and polynucleated cells. **(a)** Frequency histogram obtained by the sorting of WT (top) and R219H (bottom) hiPSC–CM. **(b)** Histogram reporting the fraction of polynucleated hiPSC–CM cells after 20, 30 and 60 days of differentiation (D20, D30 D60). Regardless of the day of differentiation, R219H hiPSC–CM always displayed a reduced fraction of polynucleated cells when compared to WT hiPSC–CM. The fraction of R219H polynucleated cells tend to decrease with the differentiation duration. **(c)**. The hiPSC–CM cell size (forward scatter, FSC) and granularity (side scatter, SSC) were analyzed for mononucleated (left) and polynucleated (right). For each panel, the first row shows the dots plot representing all events,

the middle row indicates the frequency histogram of cells size while the bottom row represents the frequency histogram of cell granularity. For each panel, the first, second and third columns respectively represent hiPSC–CM after 20, 30 and 60 days of differentiation (D20, D30 and D60).

Figure S8: Relative hiPSC–CM expression of several potassium and calcium channels

Relative quantities versus RPL22 for each sample of K_{ir}, K_v7.1, K_v11.1, MinK, Ca_v1.2 and Ca_v1.3 transcripts evaluated by real–time reverse transcription polymerase chain reaction (qRT–PCR) using mRNA extract from hiPSC–CM at day 60 of differentiation. The up–regulation of almost all transcripts has been attributed to the increased cell size observed using the cell sorter and is consistent with other qRT–PCR and western blot results shown in Fig. S3.

Figure S9: Effect of stimulation frequency on AP properties

(a - d) Examples of APs elicited at different stimulation frequency of ventricular like cells **(a, c)** and atrial like cells **(b, d). (e - i)**. $APD_{90–50–20}$, the dV/dt and the overshoot are summarized as a function of the stimulation frequency for both hiPSC–CM WT and R219H. (**j**) Arrhythmic pattern (delayed after depolarizations, DADs) were recorded in gap–free mode (current clamp) for hiPSC–CM R219H auricular like cell. Black arrows indicate DADs.

Figure S10: Locations of VSDs mutations on voltage gated ion channels that may potentially be associated with the creation of gating pores

The 24 TM or 6 TM segments are shown on a 2–D representative structure. Mutations have been reported on the VSDs of several K_v channels (K_v3.1, 3.3, 7.2, 10.1), Na_v channels (Na_v1.1, 1.2, 1.4, 1.5, 1.6, 1.9) and Ca_v channels (Ca_v1.1, 2.1). These mutations are associated with the development of diseases such as ataxia, peripheral nerve hyperexcitability, epilepsies, periodic paralysis (PP, HypoPP, NormoPP), malignant hyperthermia, familial hemiplegic migraine (FHM), familial episodic pain and arrhythmias associated with DCM.

Figure S11: Full-length blot

The presence of $Na_v1.5$ protein in hiPSC–CM is confirmed by western blot experiments. $Na_v1.5$ is detected in both WT and mutant hiPSC–CM (D_{30}) while $Na_v1.5$ protein is not detected in hiPSC (D_0). NaK ATPase is also present. The β–actin expression appears stable.

References

1 Gosselin-Badaroudine, P. *et al.* A proton leak current through the cardiac sodium channel is linked to mixed arrhythmia and the dilated cardiomyopathy phenotype. *PLoS ONE* **7**, e38331, doi:10.1371/journal.pone.0038331 (2012).

2 Burridge, P. W. *et al.* Chemically defined generation of human cardiomyocytes. *Nat. Methods* **11**, 855-860, doi:10.1038/nmeth.2999 (2014).

3 Lian, X. *et al.* Robust cardiomyocyte differentiation from human pluripotent stem cells via temporal modulation of canonical Wnt signaling. *Proc Natl Acad Sci U S A* **109**, E1848-1857, doi:10.1073/pnas.1200250109 (2012).

4 Lian, X. *et al.* Directed cardiomyocyte differentiation from human pluripotent stem cells by modulating Wnt/beta-catenin signaling under fully defined conditions. *Nat. Protoc.* **8**, 162-175, doi:10.1038/nprot.2012.150 (2013).

5 Mercier, A. *et al.* Nav1.5 channels can reach the plasma membrane through distinct N-glycosylation states. *Biochim. Biophys. Acta* **1850**, 1215-1223, doi:10.1016/j.bbagen.2015.02.009 (2015).

6 Moreau, A., Gosselin-Badaroudine, P., Delemotte, L., Klein, M. L. & Chahine, M. Gating pore currents are defects in common with two Nav1.5 mutations in patients with mixed arrhythmias and dilated cardiomyopathy. *J Gen Physiol* **145**, 93-106, doi:10.1085/jgp.201411304 (2015).

7 Payandeh, J., Scheuer, T., Zheng, N. & Catterall, W. A. The crystal structure of a voltage-gated sodium channel. *Nature* **475**, 353-358, doi:10.1038/nature10238 (2011).

8 Sievers, F. *et al.* Fast, scalable generation of high-quality protein multiple sequence alignments using Clustal Omega. *Mol. Syst. Biol.* **7**, 539, doi:10.1038/msb.2011.75 (2011).

9 Eswar, N. *et al.* Comparative protein structure modeling using Modeller. *Current protocols in bioinformatics / editoral board, Andreas D. Baxevanis ... [et al.]* **Chapter 5**, Unit 5 6, doi:10.1002/0471250953.bi0506s15 (2006).

10 Gosselin-Badaroudine, P., Delemotte, L., Moreau, A., Klein, M. L. & Chahine, M. Gating pore currents and the resting state of Nav1.4 voltage sensor domains. *Proc Natl Acad Sci U S A* **109**, 19250-19255, doi:10.1073/pnas.1217990109 (2012).

11 Wood, M. L. *et al.* Water wires in atomistic models of the Hv1 proton channel. *Biochim. Biophys. Acta* **1818**, 286-293, doi:10.1016/j.bbamem.2011.07.045 (2012).

12 Guo, A. & Song, L. S. AutoTT: automated detection and analysis of T-tubule architecture in cardiomyocytes. *Biophys J* **106**, 2729-2736, doi:10.1016/j.bpj.2014.05.013 (2014).

Table S1. Clinical phenotype of the index patient carrying the Na_v1.5/R219H mutation

	Initial description	Last follow up
**Rhythm disturbances**		
Atrial	AFL, ISR	PVD
Conduction system	AVB I, AVB III, delayed AH (145ms)	
Ventricular	VT, PVD, nQRS	
Other	Bradycardia, palpitations	
**Heart morphology**		
LVEDD (mm)	62	61
LVESD (mm)		46
LVEF (%)	49	49
LA (mm)	43	46
LAVI (ml/m^2)		44
IVSd (mm)		10
PWd (mm)		9
**Treatment**		
	DDD Pacemaker / ICD	
	ACE-inhibitor (perindopril)	
	Diuretic (Indapamid)	
	Beta-blocker (bisoprolol)	

AF, atrial fibrillation; **AFL,** Atrial flutter; **AVB I/III,** $1^{st}/3^{rd}$ degree atrio-ventricular block; **ICD,** implantable cardioverter defibrillator; **ISR,** intermittent sinus rhythm; **IVSd,** inter-ventricular septum diastolic; **LA,** left atrium; **LAVI,** left atrium volume index; **LVEDD,** left ventricular end-diastolic diameter; **LVEF,** left ventricular ejection fraction; **LVESD,** left ventricular end-systolic diameter; **nQRS,** narrow QRS complex; **PVD,** premature ventricular depolarization; **PWd,** posterior wall diastolic; **VT,** ventricular tachycardia.

Table S2. Biophysical parameters of $Na_v1.5$ / WT and R219H from hiPSC-CM

	WT (n=9)	R219H (n=10)
**Peak Current (pA/pF)**	-184.5 ± 28.3	-194.1 ± 26.7
**Activation**		
$V_{1/2}$ (mV)	-40.7 ± 1.2	-41.8 ± 1.9
k (mV)	-5.9 ± 0.6	-5.7 ± 0.3
**Inactivation**		
$V_{1/2}$ (mV)	-83.2 ± 0.9	-81.9 ± 1.9
k (mV)	5.8 ± 0.2	5.2 ± 0.2
**Recovery from inactivation**		
A_{fast}	0.77 ± 0.03	0.82 ± 0.03
A_{slow}	0.23 ± 0.03	0.18 ± 0.03
τ_{fast} (ms)	29.3 ± 2.4	26.5 ± 3.2
τ_{slow} (ms)	248.4 ± 40	324.7 ± 54.5
**Peak Ramp Current (% peak current)**	5.6 ± 0.7	5.6 ± 0.4
**Persistent Current (% peak current)**	0.38 ± 0.1	0.35 ± 0.1

$V_{1/2}$ - Mid point for activation or inactivation.

k - Slow factor for activation or inactivation.

Table S3. FACS parameters of hiPSC-CM WT and R219H

		Mononucleated		Polynucleated	
		**WT**	**R219H**	**WT**	**R219H**
D20	Percentage (%)	40.4 ± 8.1	55.8 ± 3.6	59.6 ± 8.1	44.2 ± 3.6
	Size (FFC)	26387 ± 1285	25096 ± 1155	122159 ± 9013	118079 ± 6665
	Granularity (SSC)	24510 ± 1289	24750 ± 867	79812 ± 4890	101598 ± 8148
D30	Percentage (%)	35.8 ± 3.8	63.8 ± 1.2**	64.2 ± 3.8	36.2 ± 1.2**
	Size (FFC)	25927 ± 1616	46106 ± 4243*	118236 ± 2445	141066 ± 1862**
	Granularity (SSC)	23162 ± 2118	46693 ± 3279**	74189 ± 5671	137680 ± 5640**
D60	Percentage (%)	45.8 ± 1.4	73.2 ± 0.6***	54.2 ± 1.4	26.8 ± 0.6***
	Size (FFC)	29582 ± 3362	49575 ± 2874*	131400 ± 689	138830 ± 888**
	Granularity (SSC)	29146 ± 2827	50827 ± 2452**	97036 ± 3202	128309 ± 2372**

Table S4. Action potential parameters of hiPSC-CM WT and R219H

		hiPSC–CM WT		hiPSC–CM / R219H	
		Ventricular	Auricular	Ventricular	Auricular
**RMP, mV (n)**		-70.0 ± 1.8 (10)	-61.3 ± 1.5 (8)	-60.1 ± 1.3 (10)***	-61.5 ± 1.9 (7)
**APD, ms (n)**					
20	0.5 Hz	229.8 ± 23.8 (8)	33.7 ± 8 (8)	300.5 ± 34.3 (12)*	118.1 ± 22.3 (12)***
	0.8 Hz	191.9 ± 9.9 (10)	29.2 ± 5.6 (9)	238.0 ± 19.1 (12)	97.0 ± 15.8 (11)***
	1 Hz	170.9 ± 11 (10)	29.2 ± 5.3 (9)	227.4 ± 18.8 (13)*	88.6 ± 12.7 (11)**
	1.3 Hz	147.3 ± 14.5 (8)	23.7 ± 4.9 (7)	171.0 ± 24.5 (7)	74.0 ± 15 (10)*
	1.5 Hz	137.2 ± 14 (8)	19.8 ± 5.4 (7)	117.4 ± 25.1 (6)	62.6 ± 15.6 (10)*
	2 Hz	95.8 ± 17 (8)	16.9 ± 4.1 (7)		54.4 ± 14.2 (9)
50	0.5 Hz	352.9 ± 30.1 (8)	73.8 ± 13.1 (8)	470.6 ± 37.0 (12)**	199.5 ± 31.1 (12)***
	0.8 Hz	292.8 ± 12.6 (10)	67.5 ± 9 (9)	371.5 ± 20.2 (12)*	162.4 ± 22.2 (11)**
	1 Hz	259.3 ± 15.7 (10)	65.9 ± 8.9 (9)	352.2 ± 22.4 (13)**	147.5 ± 16.8 (11)**
	1.3 Hz	236 ± 19.8 (8)	56.5 ± 11.2 (7)	278.1 ± 33.9 (7)	125.7 ± 22.1 (10)*
	1.5 Hz	227.1 ± 16.2 (8)	50.4 ± 13 (7)	194.1 ± 31.2 (6)	110.0 ± 23.3 (10)
	2 Hz	170.0 ± 23.8 (8)	45.8 ± 13.5 (7)		98.5 ± 21.8 (9)
90	0.5 Hz	438.1 ± 32.3 (8)	140.9 ± 12.2 (8)	571.0 ± 32.2 (12)***	268.7 ± 33.7 (12)***
	0.8 Hz	383 ± 17.9 (10)	135.3 ± 14.1 (9)	451.1 ± 23.3 (12)*	236.5 ± 24.8 (11)**
	1 Hz	345.9 ± 19.2 (10)	128.8 ± 13.7 (9)	434.2 ± 24.8 (13)*	218.7 ± 18.6 (11)*
	1.3 Hz	322.6 ± 21.8 (8)	119.5 ± 17.1 (7)	359.2 ± 35.9 (7)	196.1 ± 28.2 (10)*
	1.5 Hz	316.7 ± 20.1 (8)	113.4 ± 22.7 (7)	277.9 ± 30.5 (6)	184.2 ± 30.0 (10)
	2 Hz	263.3 ± 30.9 (8)	110.2 ± 25.9 (7)		168.2 ± 32.7 (9)
**dV/dt_{max}, mV/ms (n)**					
	0.5 Hz	47.5 ± 9.7 (8)	83.1 ± 19. 1 (8)	82.9 ± 12.5 (12)*	74.2 ± 11.8 (12)
	0.8 Hz	37.7 ± 8.4 (10)	78.4 ± 19.0 (9)	84.8 ± 9.3 (12)***	65.4 ± 14.8 (11)
	1 Hz	43.8 ± 9.6 (8)	72.0 ± 15.9 (8)	81.1 ± 8.6 (13)**	67.2 ± 15.6 (11)
	1.3 Hz	33.7 ± 6.6 (8)	49.3 ± 8.9 (7)	54.5 ± 12.6 (7)	52.4 ± 12.6 (10)
	1.5 Hz	30.4 ± 6.4 (8)	52.8 ± 10.0 (7)	68.2 ± 12.2 (6)*	70.7 ± 15.3 (10)
	2 Hz	28.3 ± 8.9 (8)	47.1 ± 10.7 (7)		67.6 ± 18.1 (9)
**Overshoot, mV (n)**					
	0.5 Hz	41.8 ± 2.9 (8)	33.8 ± 4.2 (8)	51.6 ± 1.6 (12)*	38.5 ± 4.1 (12)
	0.8 Hz	39.4 ± 2.5 (10)	31.7 ± 5.2 (9)	48.2 ± 2.0 (12)*	37.2 ± 4.9 (11)
	1 Hz	38.3 ± 2.8 (10)	29.4 ± 5.1 (9)	49.7 ± 1.7 (13)**	37.6 ± 5.2 (11)
	1.3 Hz	33.8 ± 4 (8)	31.5 ± 5.4 (7)	46.3 ± 2.9 (7)**	33.6 ± 5.2 (10)
	1.5 Hz	33 ± 3.7 (8)	31.6 ± 5.5 (7)	41.8 ± 1.8 (6)	34.1 ± 5.5 (10)
	2 Hz	24.4 ± 5.7(8)	27.6 ± 7 (7)		33.2 ± 5.5 (9)

Table S5. Atomic force microscopy parameters of hiPSC-CM WT and R219H

	WT (n=24)	R219H (n=40)
**Amplitude (nN)**	1.31 ± 0.2	0.57 ± 0.07 ***
**Frequency (Hz)**	0.43 ± 0.03	1.23 ± 0.1 ***
**Contraction slope (nN/s)**	5.1 ± 0.9	4.6 ± 0.5
**Contraction duration (ms)**		
at 20 % of relaxation	153 ± 17	62 ± 7 ***
at 50 % of relaxation	275 ± 30	119 ± 13 ***
at 90 % of relaxation	499 ± 63	210 ± 22 ***

* Difference between hiPSC-CM WT (***: $p < 0.001$).

Table S6. DNA primers used in the study

Protein	Primers	Length (bp)
**Na_v1.5 (cDNA)**	F – GCATACACAACTGAATTTGTGG	104
(NM_198056.2)	R – GTCTTCAGCCCTGAAATGAC	
**Na_v1.5 (DNA)**	F – GTTAGGAGGGTTGAAATCCAGAAGAAAAC	714
	R – GGTGTCTATGAGAGTGGGCTTTGCT	
**RPL22**	F – CCATGGCTCCTGTGAAAAAG	219
(NM_000983.3)	R – TCACGGTGATCTTGCTCTTG	
**GATA4**	F – TCCCTCTTCCCTCCTCAAAT	194
(NM_002052.3)	R – TCAGCGTGTAAAGGCATCTG	
**cTnT**	F – ACCAGAAAGTCTCCAAGACCC	87
(NM_000364)	R – GGAGCAGATCTTTGGTGAAGG	
**K_{ir}2.1**	F – ACCGCTACAGCATCGTCTCT	199
(NM_000891)	R – TCCACACACGTGGTGAAGAT	
**K_{ir}2.2**	F – AGCTGAGGCAGAGAGCAGAG	171
(NM_021012)	R – CGGGTGTCTTGACTTCCATT	
**K_{ir}2.3**	F – ACCTCAACGTGGGCTATGAC	152
(NM_152868)	R – CTCCAGGATGACCACGATCT	
**K_{ir}2.4**	F – GCTAAGGAGCTGGATGAACG	205
(NM_013348.3)	R – CCAGGGTTGGTGTGAGAACT	
**K_v11.1**	F – TCCATCAAGGACAAGTATGTGAC	142
(NM_000238)	R – CATACATGAGGGAGCCAATGAG	
**K_v7.1**	F – CAGTTCTGTAAGGAAGAGCCC	130
(NM_000218)	R – CTGTGAGATGTGGGTGATGG	
**MinK**	F – AGGATATTCAGAGGTGTGCCTG	105
(NM_000219)	R – TTGGTCAGAAAGGGCGTCAC	
**Ca_v1.2**	F – CAGAAACTACAGGAGAAGAGGA	207
(NM_000719)	R – AAGAAGAGGATCAGGTTGGT	
**Ca_v1.3**	F – CAATGGCACGGAATGTAGGA	148
(NM_000720)	R – TATCGCATCATTTACCCAGTAGAG	

Fig. S1

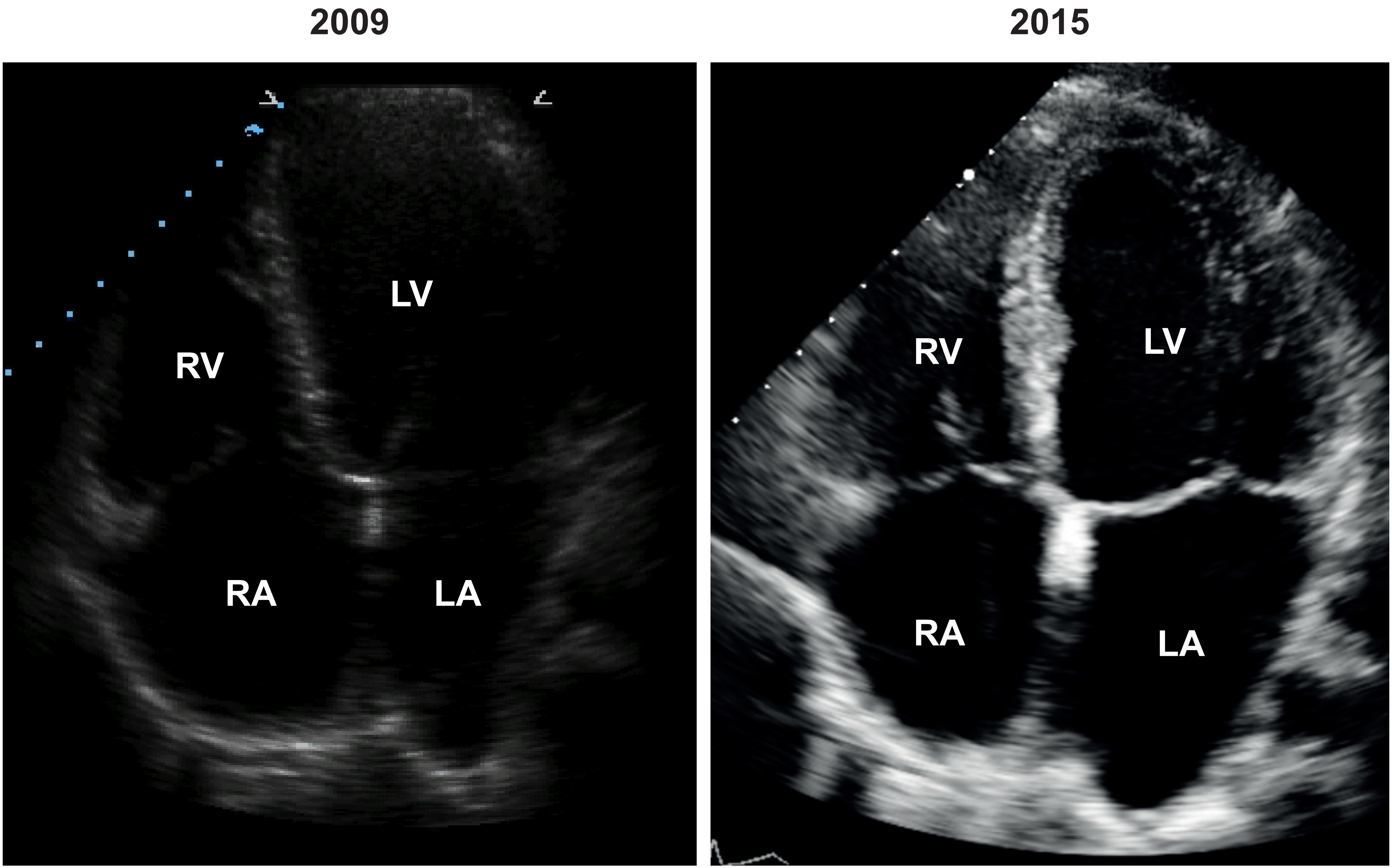

Fig. S2

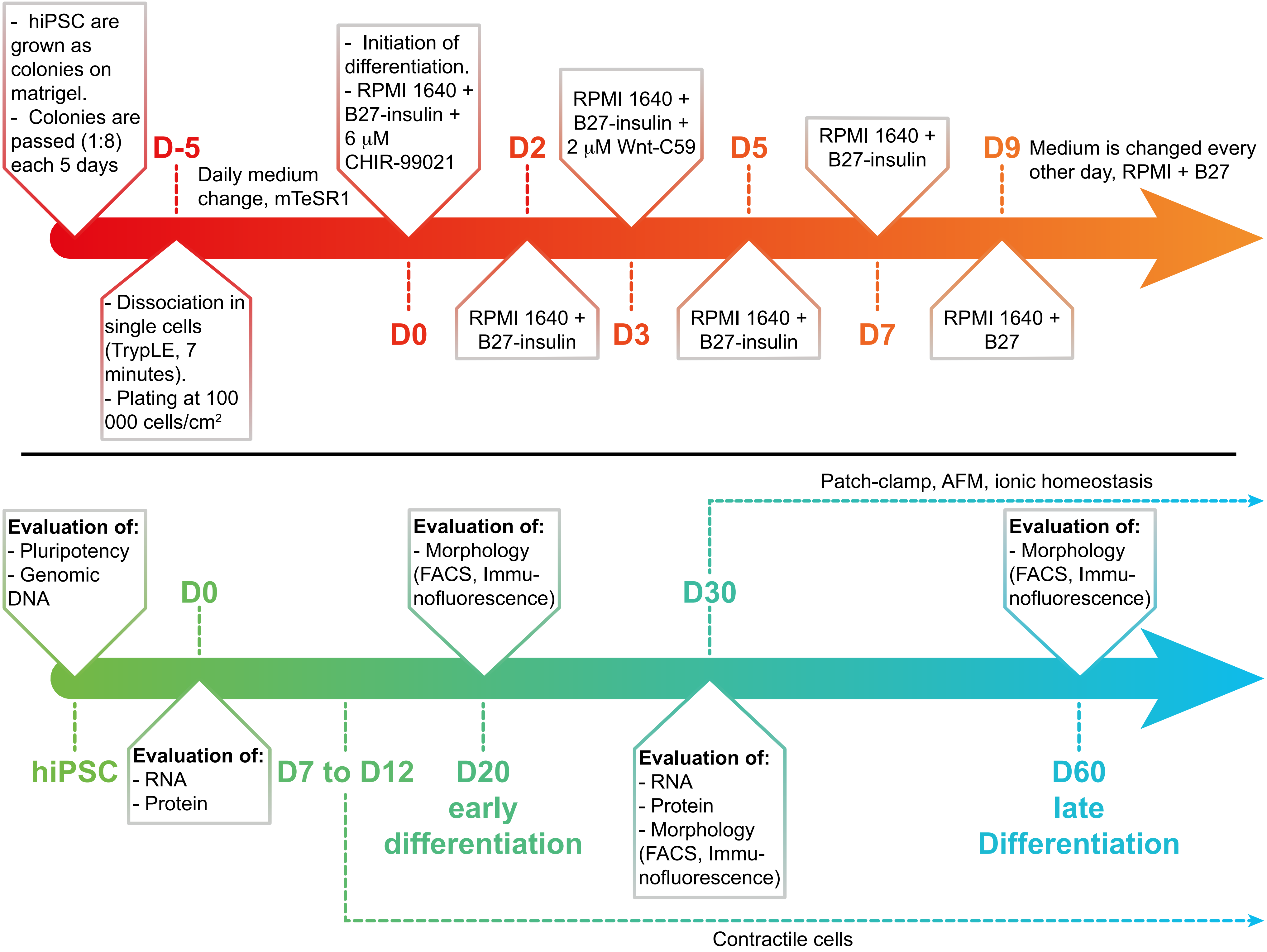

- hiPSC are grown as colonies on matrigel.
- Colonies are passed (1:8) each 5 days
D-5
Daily medium change, mTeSR1
- Dissociation in single cells (TrypLE, 7 minutes).
- Plating at 100 000 cells/cm²
- Initiation of differentiation.
- RPMI 1640 + B27-insulin + 6 µM CHIR-99021
D0
D2
RPMI 1640 + B27-insulin
RPMI 1640 + B27-insulin + 2 µM Wnt-C59
D3
D5
RPMI 1640 + B27-insulin
RPMI 1640 + B27-insulin
D7
D9
Medium is changed every other day, RPMI + B27
RPMI 1640 + B27
Patch-clamp, AFM, ionic homeostasis
Evaluation of:
- Pluripotency
- Genomic DNA
hiPSC
D0
Evaluation of:
- RNA
- Protein
D7 to D12
Evaluation of:
- Morphology (FACS, Immu-nofluorescence)
D20
early
differentiation
D30
Evaluation of:
- RNA
- Protein
- Morphology (FACS, Immu-nofluorescence)
Evaluation of:
- Morphology (FACS, Immu-nofluorescence)
D60
late
Differentiation
Contractile cells

Fig. S3

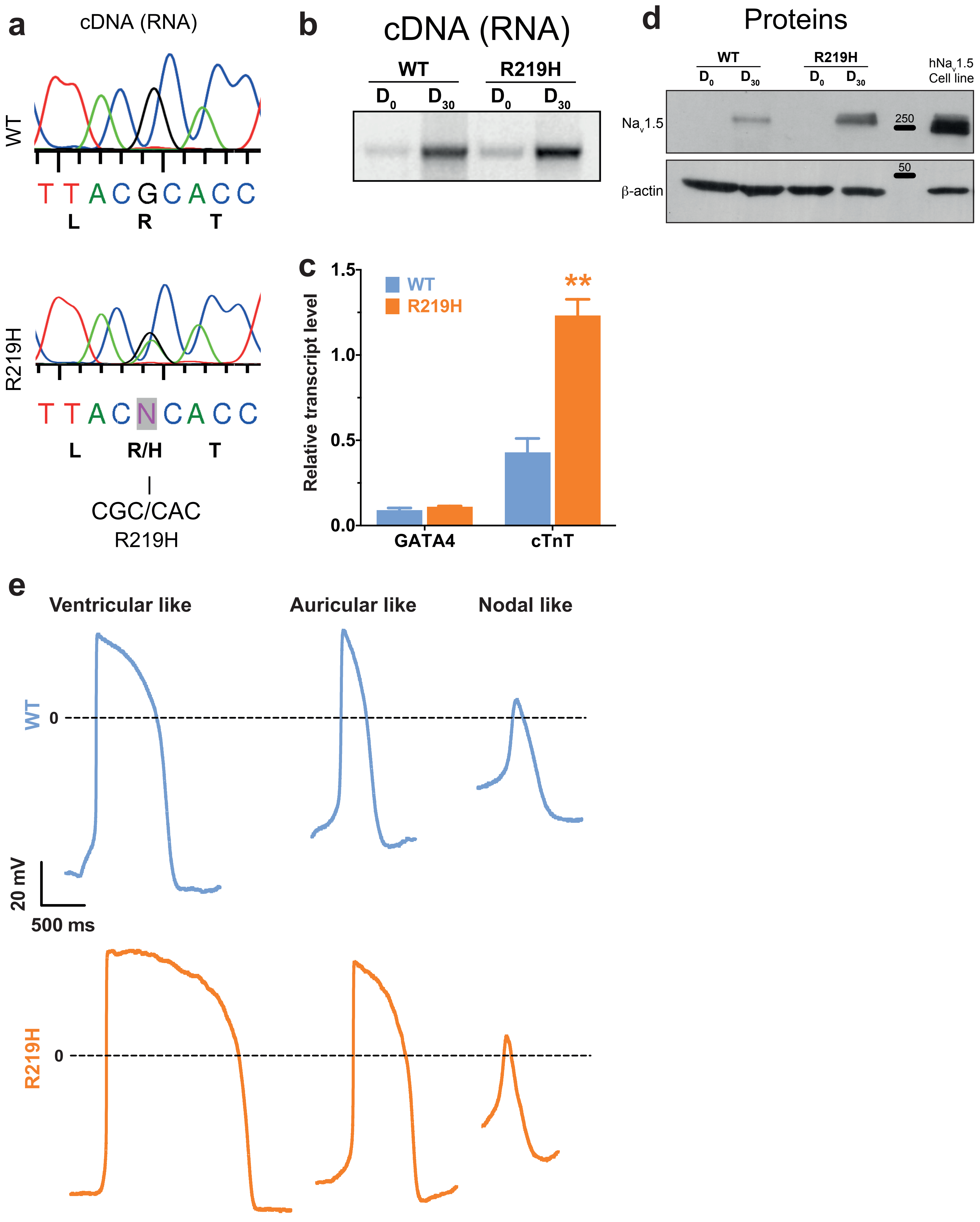

a
cDNA (RNA)
WT
T T A C G C A C C
L R T
R219H
T T A C N C A C C
L R/H T
CGC/CAC
R219H
b
cDNA (RNA)
WT
R219H
D0 D30 D0 D30
c
Relative transcript level
1.5
1.0
0.5
0.0
WT
R219H
**
GATA4
cTnT
d
Proteins
WT
R219H
D0 D30 D0 D30
hNav1.5
Cell line
Nav1.5
250
50
β-actin
e
Ventricular like
Auricular like
Nodal like
WT
0
20 mV
500 ms
R219H
0

Fig. S4

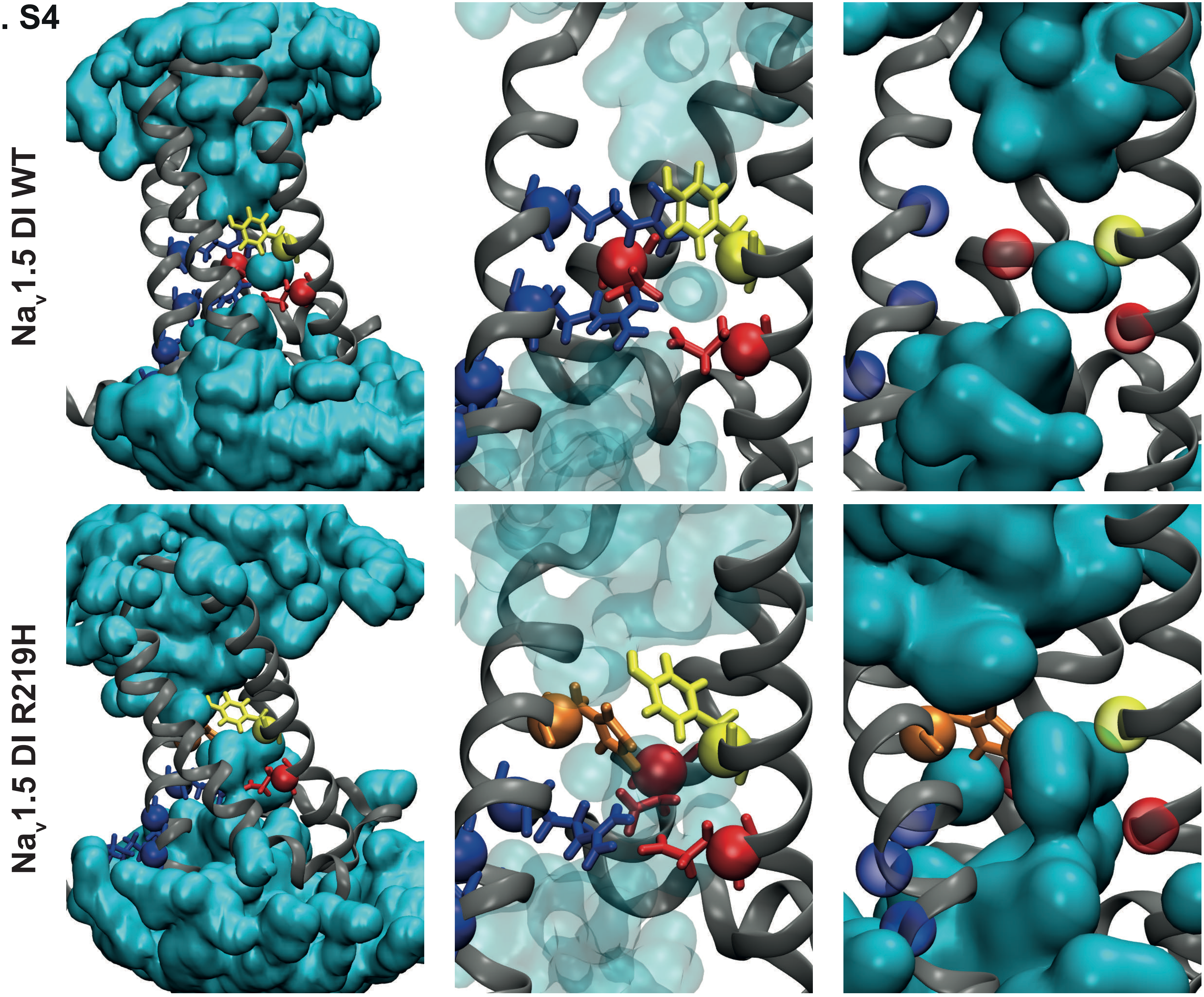

Fig. S5

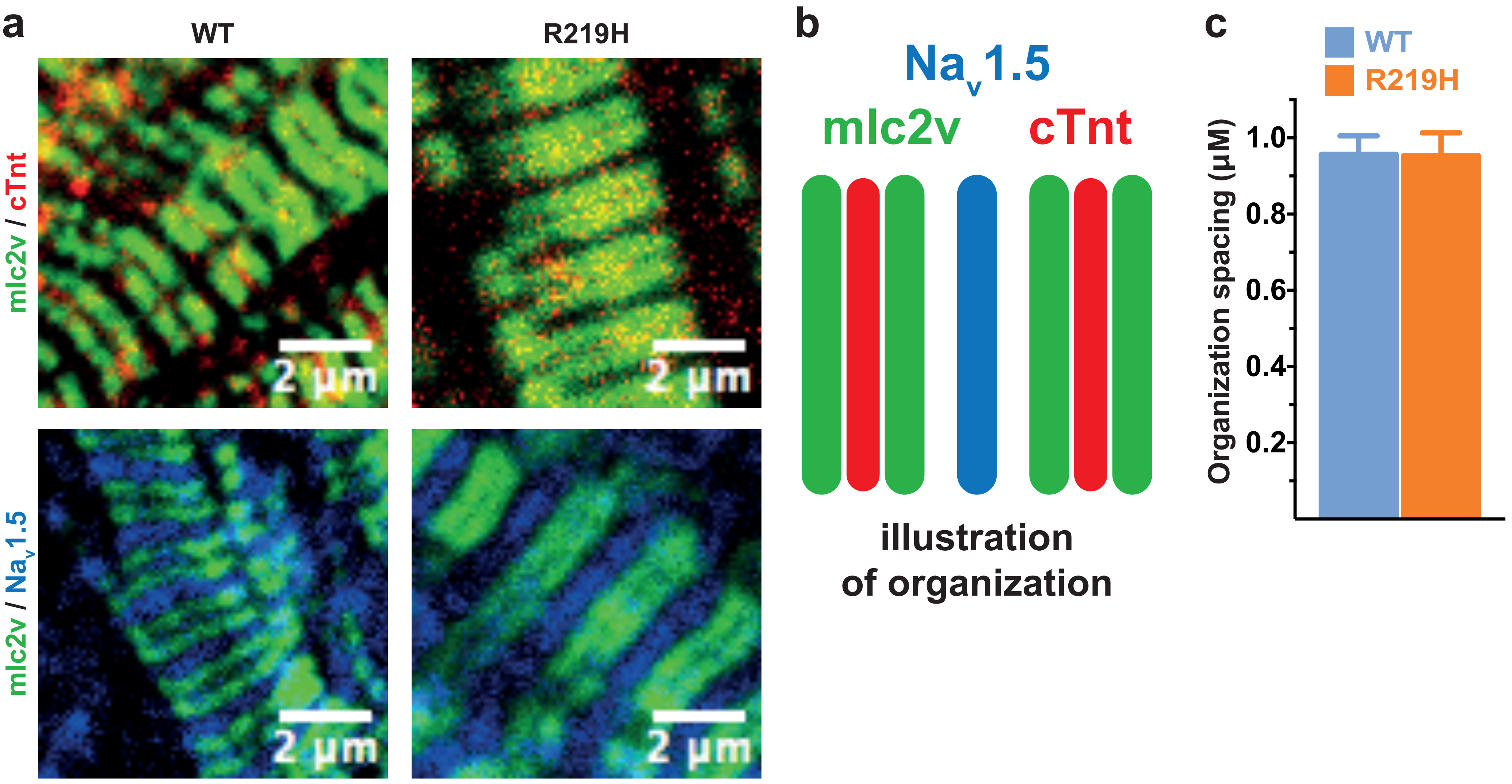
a
WT
R219H
mlc2v / cTnt
mlc2v / $Na_v1.5$
2 µm
2 µm
2 µm
2 µm
b
$Na_v1.5$
mlc2v
cTnt
illustration
of organization
c
WT
R219H
Organization spacing (µM)
1.0
0.8
0.6
0.4
0.2

Fig. S6

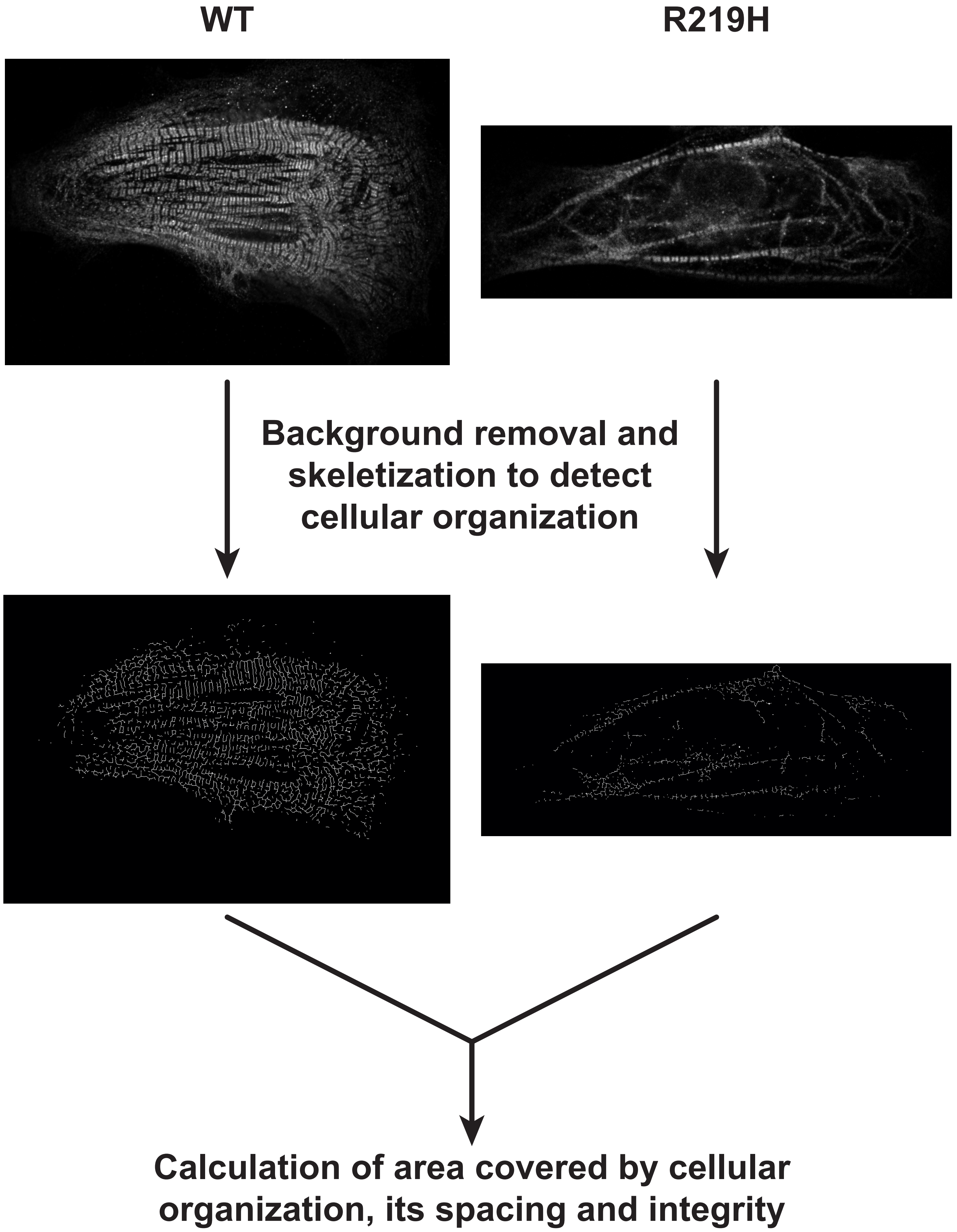
WT
R219H
Background removal and
skeletization to detect
cellular organization
Calculation of area covered by cellular
organization, its spacing and integrity

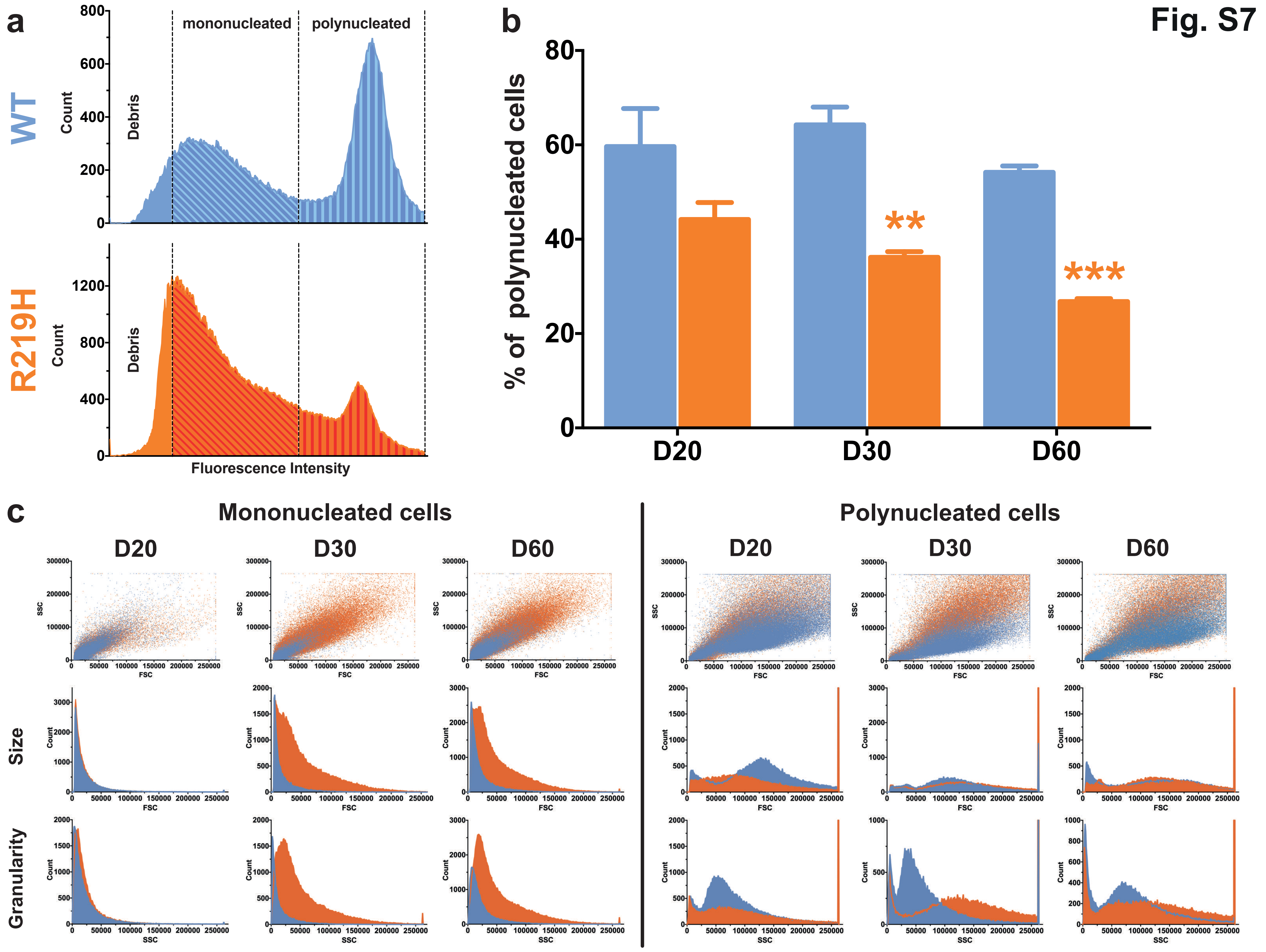
Fig. S7
a
WT
R219H
Count
Debris
mononucleated
polynucleated
Fluorescence Intensity
b
% of polynucleated cells
D20
D30
D60
**
***
c
Mononucleated cells
Polynucleated cells
D20
D30
D60
Size
Granularity
SSC
FSC
Count

Fig. S8

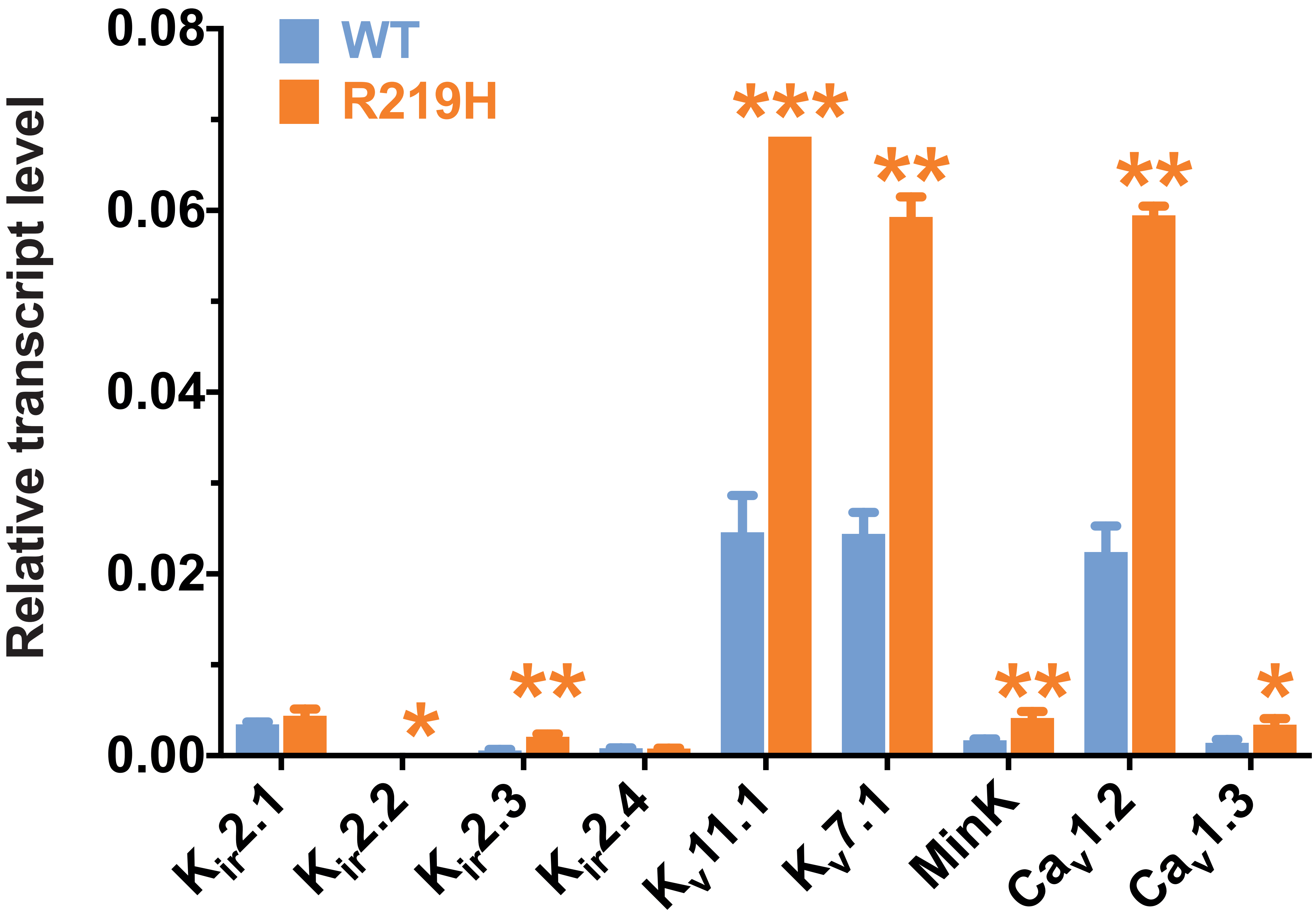

WT
R219H
Relative transcript level
0.08
0.06
0.04
0.02
0.00
***
**
**
**
**
*
*
$K_{ir}2.1$
$K_{ir}2.2$
$K_{ir}2.3$
$K_{ir}2.4$
$K_v11.1$
$K_v7.1$
MinK
$Ca_v1.2$
$Ca_v1.3$

Fig. S9

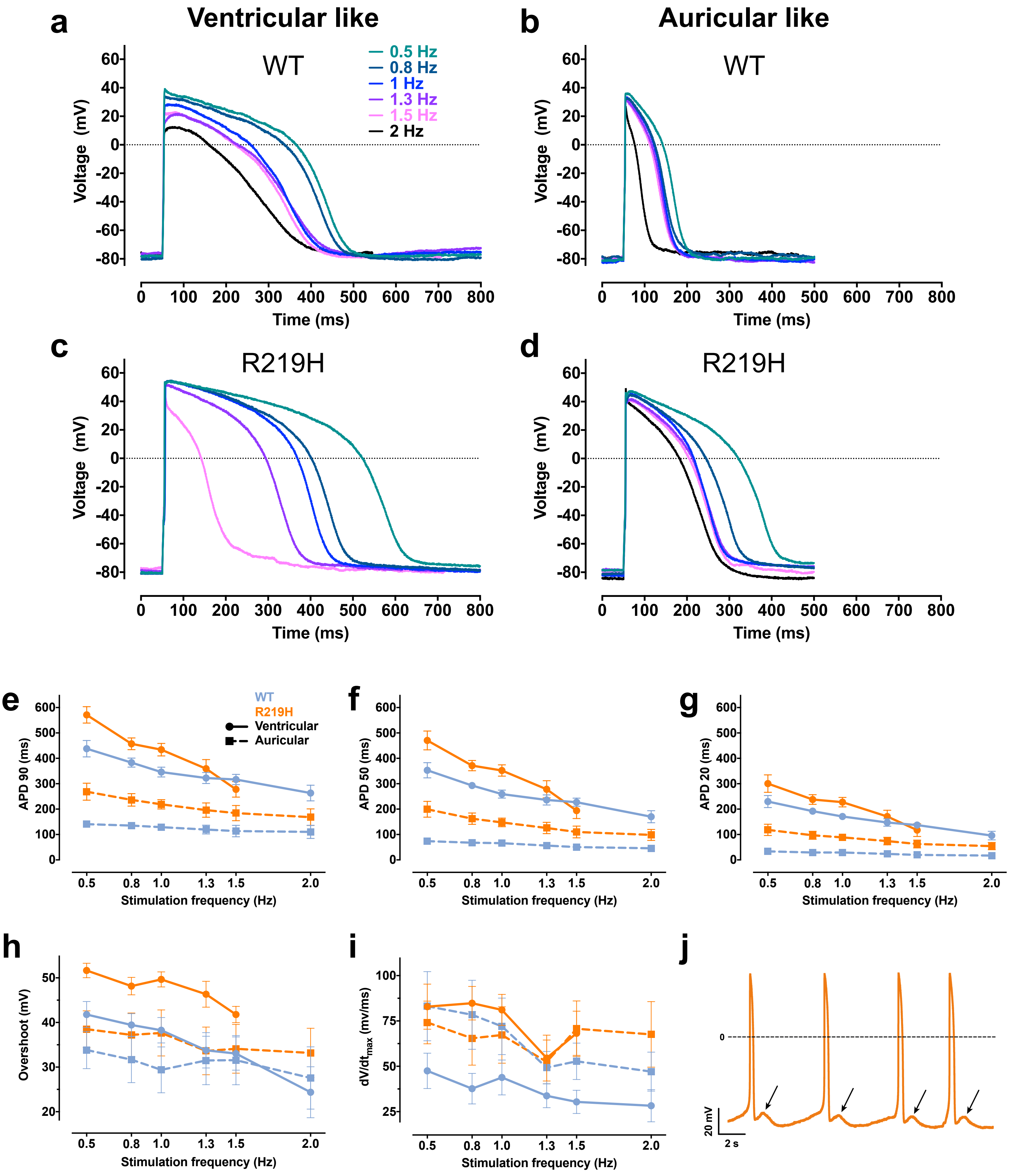

a
Ventricular like
b
Auricular like
WT
0.5 Hz
0.8 Hz
1 Hz
1.3 Hz
1.5 Hz
2 Hz
Voltage (mV)
Time (ms)
c
d
R219H
e
f
g
WT
R219H
Ventricular
Auricular
APD 90 (ms)
APD 50 (ms)
APD 20 (ms)
Stimulation frequency (Hz)
h
i
j
Overshoot (mV)
dV/dt max (mv/ms)
0
20 mV
2 s

Fig. S10

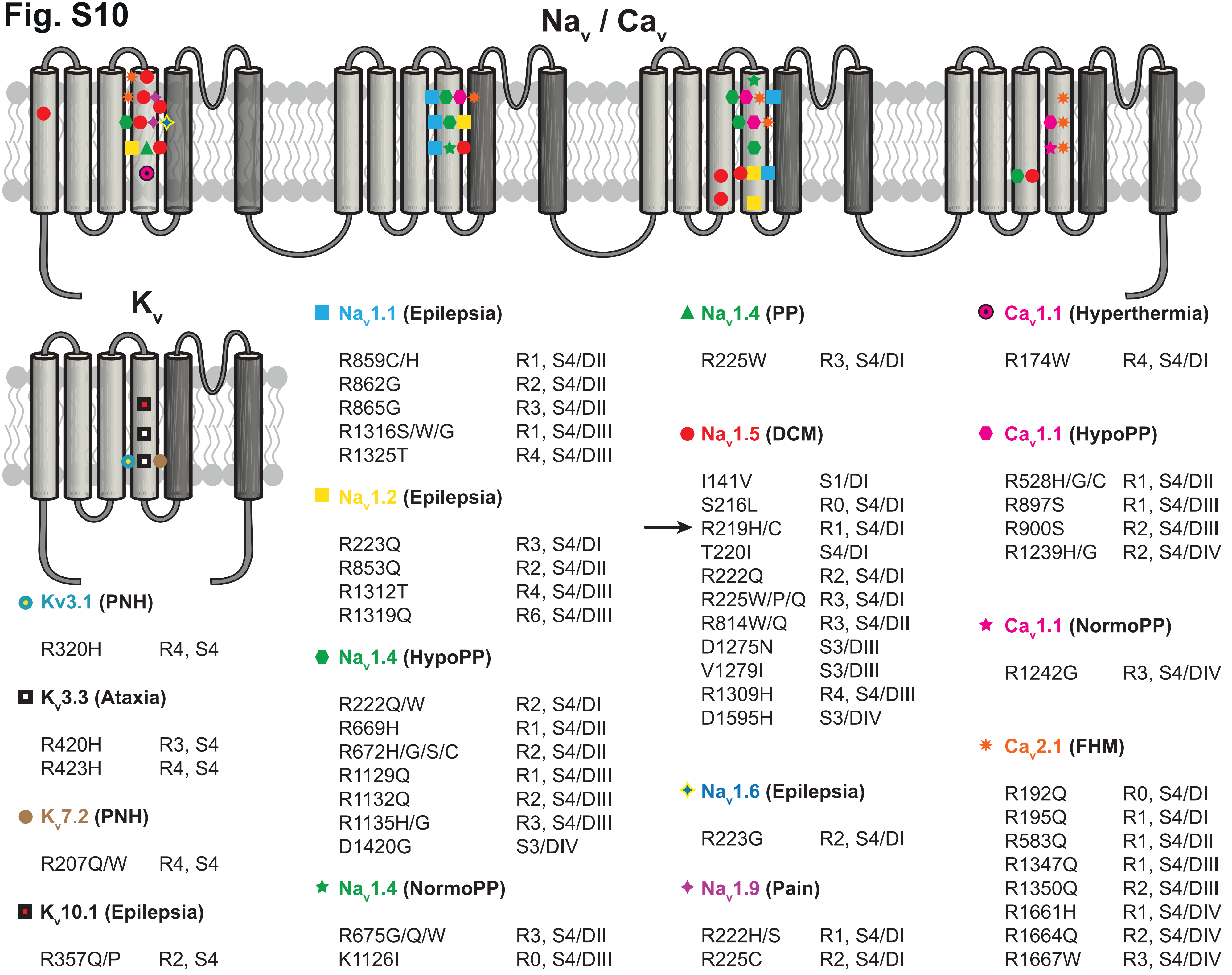

Na$_v$ / Ca$_v$
K$_v$
Na$_v$1.1 (Epilepsia)
R859C/H R1, S4/DII
R862G R2, S4/DII
R865G R3, S4/DII
R1316S/W/G R1, S4/DIII
R1325T R4, S4/DIII
Na$_v$1.2 (Epilepsia)
R223Q R3, S4/DI
R853Q R2, S4/DII
R1312T R4, S4/DIII
R1319Q R6, S4/DIII
Na$_v$1.4 (HypoPP)
R222Q/W R2, S4/DI
R669H R1, S4/DII
R672H/G/S/C R2, S4/DII
R1129Q R1, S4/DIII
R1132Q R2, S4/DIII
R1135H/G R3, S4/DIII
D1420G S3/DIV
Na$_v$1.4 (NormoPP)
R675G/Q/W R3, S4/DII
K1126I R0, S4/DIII
Na$_v$1.4 (PP)
R225W R3, S4/DI
Na$_v$1.5 (DCM)
I141V S1/DI
S216L R0, S4/DI
R219H/C R1, S4/DI
T220I S4/DI
R222Q R2, S4/DI
R225W/P/Q R3, S4/DI
R814W/Q R3, S4/DII
D1275N S3/DIII
V1279I S3/DIII
R1309H R4, S4/DIII
D1595H S3/DIV
Na$_v$1.6 (Epilepsia)
R223G R2, S4/DI
Na$_v$1.9 (Pain)
R222H/S R1, S4/DI
R225C R2, S4/DI
Ca$_v$1.1 (Hyperthermia)
R174W R4, S4/DI
Ca$_v$1.1 (HypoPP)
R528H/G/C R1, S4/DII
R897S R1, S4/DIII
R900S R2, S4/DIII
R1239H/G R2, S4/DIV
Ca$_v$1.1 (NormoPP)
R1242G R3, S4/DIV
Ca$_v$2.1 (FHM)
R192Q R0, S4/DI
R195Q R1, S4/DI
R583Q R1, S4/DII
R1347Q R1, S4/DIII
R1350Q R2, S4/DIII
R1661H R1, S4/DIV
R1664Q R2, S4/DIV
R1667W R3, S4/DIV
Kv3.1 (PNH)
R320H R4, S4
K$_v$3.3 (Ataxia)
R420H R3, S4
R423H R4, S4
K$_v$7.2 (PNH)
R207Q/W R4, S4
K$_v$10.1 (Epilepsia)
R357Q/P R2, S4

Fig. S11

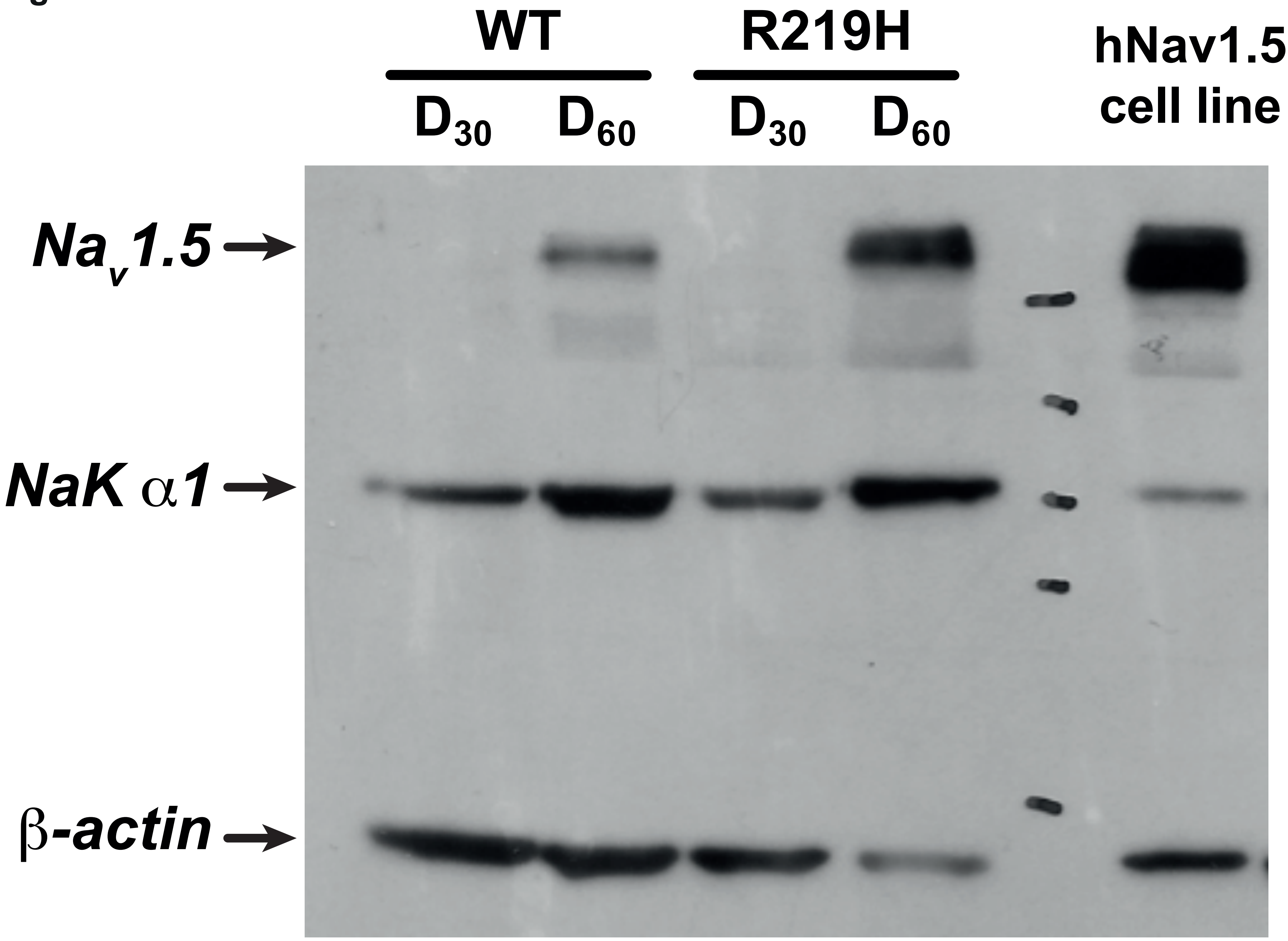

Supplement: Supplementary file 1 — Supplemental Data [file 41598_2018_31772_MOESM1_ESM.pdf]
